# Insights into Functions of Universal Stress Proteins Encoded by Genomes of Gastric Cancer Pathogen *Helicobacter pylori* and Related Bacteria

**DOI:** 10.3390/pathogens14030275

**Published:** 2025-03-13

**Authors:** Raphael D. Isokpehi, Shaneka S. Simmons, Angela U. Makolo, Antoinesha L. Hollman, Solayide A. Adesida, Olabisi O. Ojo, Amos O. Abioye

**Affiliations:** 1 Transdisciplinary Data Scholars Development Program, Bethune-Cookman University, Daytona Beach, FL 32114, USA; 2 Division of Arts and Sciences, Jarvis Christian University, Hawkins, TX 75765, USA; 3 University of Ibadan Bioinformatics Group, Department of Computer Science, University of Ibadan, Ibadan 200005, Oyo State, Nigeria; 4 Department of Microbiology, Faculty of Science, University of Lagos, Akoka 101017, Lagos State, Nigeria; 5 Department of Natural Sciences, Albany State University, Albany, GA 31721, USA; 6 College of Pharmacy & Health Sciences, Belmont University, Nashville, TN 37212, USA; amos.abioye@belmont.edu

**Keywords:** ATP-binding, bioinformatics, DNA damage, DNA uptake, environmental stress response, gastric cancer, genomic context, *Helicobacteracae*, *Helicobacter pylori*, natural transformation competence, universal stress proteins, virulence, *Wolinella*

## Abstract

The genes that encode the universal stress protein (USP) family domain (pfam00582) aid the survival of bacteria in specific host or habitat-induced stress conditions. Genome sequencing revealed that the genome of *Helicobacter pylori*, a gastric cancer pathogen, typically contains one USP gene, while related helicobacters have one or two distinct USP genes. However, insights into the functions of *Helicobacteraceae* (*Helicobacter* and *Wolinella*) USP genes are still limited to inferences from large-scale genome sequencing. Thus, we have combined bioinformatics and visual analytics approaches to conduct a more comprehensive data investigation of a set of 1045 universal stress protein sequences encoded in 1014 genomes including 785 *Helicobacter pylori* genomes. The study generated a representative set of 183 USP sequences consisting of 180 *Helicobacter* sequences, two *Wolinella succinogenes* sequences, and a sequence from a related campylobacteria. We used the amino acid residues and positions of the 12 possible functional sites in 1030 sequences to identify 25 functional sites patterns for guiding studies on functional interactions of *Helicobacteraceae* USPs with ATP and other molecules. Genomic context searches and analysis identified USP genes of gastric and enterohepatic helicobacters that are adjacent or in operons with genes for proteins responsive to DNA-damaging oxidative stress (ATP-dependent proteases: ClpS and ClpA); and DNA uptake proteins (natural competence for transformation proteins: ComB6, ComB7, ComB8, ComB9, ComB10, ComBE, and conjugative transfer signal peptidase TraF). Since transcriptomic evidence indicates that oxidative stress and the presence of virulence-associated genes regulate the transcription of *H. pylori* USP gene, we recommend further research on *Helicobacter* USP genes and their neighboring genes in oxidative stress response and virulence of helicobacters. To facilitate the reuse of data and research, we produced interactive analytics resources of a dataset composed of values for variables including phylogeography of *H. pylori* strains, protein sequence features, and gene neighborhood.

## 1. Introduction

The genera in the Gram-negative bacteria family named *Helicobacteraceae* currently includes *Helicobacter*, with 55 species, and *Wolinella*, with one species [1,2]. The *Helicobacter* species are commonly associated with the gastrointestinal system of animal or human hosts, where they may be responsible for acute infections or chronic infections that could lead to malignancies including gastric cancer [3]. Helicobacters have been broadly grouped by anatomic niches as gastric (stomach) and enterohepatic (intestinal tract and liver) [4]. The zoonotic potential of avian *Helicobacter species*, such as *H. pullorum* and *H. canadensis*, is of public health concern [5]. *Helicobacter* species that infect the human gastrointestinal system must adapt and survive extreme conditions such as acidic gastric juice, antimicrobial compounds, bile, low pH, and oxidative stress from host immune response [6,7]. *Helicobacter pylori* is a microaerophilic, neutralophilic, and flagellated pathogen that infects the highly acidic human stomach [8].

We have previously investigated the functions of genes that encode the universal stress protein (Usp) domain (Pfam protein family: pfam00582 or PF00582) in bacteria and eukaryotes [9,10,11,12,13,14,15]. The universal stress proteins (USPs) are broadly classified by the presence of amino acid residues that bind to adenosine triphosphate (ATP) [16,17,18]. In both pathogenic and non-pathogenic bacteria, the USPs enhance adaptive survival in diverse unfavorable conditions including high osmolarity [19], iron scavenging [20], acid fluctuations [21], nutrient starvation [22,23], and antibiotic resistance [24,25]. ATP binding of mycobacteria universal stress proteins influences chronic persistent infection and intracellular survival [26,27].

The spectrum of clinical manifestations of infection by *Helicobacteracae* presents a need for a greater understanding of the biological processes, including response to stress by this bacteria family. However, the functions of genes for *Helicobacteraceae* universal stress proteins are still limited to data in the Supplementary Files on genome-enabled investigations of *Helicobacter pylori* strains [28,29]. For example, a study of the primary transcriptome of *H. pylori* 26695 included a supplementary operon map with the USP gene (HP0031) in a 15-gene operon [28]. Thus, the goal of our data investigation project was to obtain insights into the functional associations of *Helicobacteraceae* universal stress proteins in host organisms. To achieve this goal, we designed four data investigation objectives.

Our first objective was to construct a representative set of universal stress protein sequences predicted from a collection of *Helicobacteraceae* genomes. This representative set of sequences from multiple species/strains of *Helicobacter* and *Wolinella* would provide a reference set for further sequence and phylogenetic analysis. Our second objective was to group the *Helicobacteraceae* species according to the patterns of functional sites in the *Helicobacteraceae* USPs. This objective is significant, because identical patterns of functional sites of USPs could suggest common ancestry of *Helicobacteraceae* species. In addition, amino acid residues involved in ATP-binding could infer ATP-dependent biological processes such as growth regulation, as observed with *Mycobacterium tuberculosis* [26,27,30]. Our third objective was to determine the transcription direction and functions of genes adjacent to *Helicobacteraceae* USP genes. Findings from this objective could guide further research on *Helicobacteraceae* USPs that regulate biological processes encoded by adjacent genes. In *Mycobacterium smegmatis*, an operon containing a USP and gene for acyltransferase was implicated in regulating biofilm formation [31]. Our fourth objective was to collect transcriptome (RNA-seq) and interactome (protein–protein interactions) evidence for biological activity of *H. pylori* USP gene. This last objective will help verify or validate findings from the data investigations. Altogether, results from our objectives provided insights into functions, directions for further research, and potential applications of the universal stress proteins of *H. pylori* and related bacteria.

## 2. Methods

### 2.1. Retrieval of Helicobacteraceae Genomes Dataset and Genes Encoding Universal Stress Protein Domain Dataset

In the first stage, we retrieved a dataset of *Helicobacteraceae* genomes that included genome identifiers (such as Genome ID and Genome Name) from the Integrated Microbial Genomes & Microbiomes Expert Review (IMG/M-ER) website [32] using a uniform resource locator (URL) search script that included the bacteria family name. The Data Availability section of this report has details of the URL search script. The dataset of genomes was stored in the IMG/M-ER Genome Cart. In the second stage, we used the IMG/MER Function Search tool with Pfam list filter to retrieve a dataset of genes annotated with the universal stress protein domain (pfam00582) for the *Helicobacteraceae* genomes retrieved into the Genome Cart in the first stage. The genome and gene datasets were saved as tab-delimited files for interactive data visualization to identify subgroups and patterns [33] in Tableau Desktop and Tableau Public (Seattle, WA, USA), a visual analytics software [34].

### 2.2. Grouping of Helicobacteraceae Species by Patterns of Functional Sites in Universal Stress Protein Sequences

We retrieved the FASTA formatted amino acid sequences for the predicted *Helicobacteraceae* universal stress proteins from the IMG/M-ER bioinformatics resource. In order to predict the functional sites, the collection of sequences was submitted to the National Center for Biotechnology Information (NCBI) Batch Web Conserved Domain (CD) Search Tool [35]. The results of the NCBI CD-Search tool for protein domain features include amino acid residue letters and positions of the functional sites (ligands). The expected count of functional sites for USPs is 12 [17]. An example of functional sites coordinates with 12 functional sites is A8, T9, D10, V38, V100, G101, T103, G104, G115, S116, V117, and T118. We constructed a protein functional sites dataset for investigations in visual analytics software [34] by deleting the comment section of the protein domain features output file from NCBI CD-Search tool. We then used visual analytics software to construct calculated fields such as patterns of the sequence of the amino acid residues of functional sites and patterns of the sequence of the amino acid positions of functional sites. A combination of (1) residue at third position of the functional sites coordinate; (2) the patterns of functional sites; and (3) protein sequence length allowed us to group *Helicobacteraceae* species. We selected the third position of the functional sites pattern because of the effect on ATP binding. In *Mycobacterium tuberculosis*, a change of aspartate (D) to glutamate (E) in the third position of the functional sites pattern of two universal stress proteins (Rv2623 and Rv2624c) abrogated ATP binding [26,27].

### 2.3. Similarity of Helicobacteraceae Universal Stress Proteins Sequences

We used the Cluster Database at High Identity with Tolerance (CD-HIT) biological sequence clustering software [36], implemented by the Galaxy@Pasteur platform [37], to cluster (at 100% identity) a set of *Helicobacteraceae* universal stress protein sequences. We constructed a dataset from the list of CD-HIT clusters of sequences with spreadsheet software, Microsoft Excel, and added strain phylogeographic annotations [38]. The non-redundant set of protein sequences produced by CD-HIT were aligned on the MAFFT multiple sequence alignment software [39]. We viewed and interacted with the multiple sequence alignment using the NCBI Multiple Sequence Alignment Viewer (version 1.25.0) [40]. In addition, phylogenetic trees visualizing the multiple sequence alignment of the representative *Helicobacteraceae* USP sequences were constructed with the Interactive Tree Of Life (iTOL) online tool for phylogenetic tree display and annotation [41] and the Environment for Tree Exploration (ETE) [42].

### 2.4. Genomic Context of Genes for Universal Stress Proteins in Helicobacteraceae Genomes

Genomic context is “information gleaned from the genes surrounding a gene of interest in the chromosome” [43]. The transcription direction, start and end coordinates, as well as functional annotations of genes surrounding a gene of interest are examples of genomic context information provided by genomic sequencing projects [44]. Additionally, an operon “is a collection of genes that are co-transcribed to form a single mRNA molecule in at least some set of biologically relevant conditions [45]”. Genes in bacterial genomes with similar function or co-expression can be clustered in a genomic region and arranged as operons [46,47]. Therefore, we obtained information on the genomic context of *Helicobacteraceae* universal stress protein genes by using genomic context tools available from microbial web portals [48].

Datasets retrieved from IMG/M by providing a list of gene identifiers (Gene ID) were the source of transcription direction (inferred from genome strand); base pair distances between the USP gene and adjacent gene; and protein family (pfam domain) annotation for the genes adjacent to the USP gene. We constructed a three-digit binary number to represent the genome strand annotation (+ or −) relative of the upstream and downstream genes to the direction of the USP gene. We assigned “1” to USP, and if the adjacent gene to the USP gene is in the same transcription direction, “1” represents the transcription direction relative to the USP gene, otherwise “0” is assigned. The possible three-digit representations and transcription direction using greater than and less than symbols are “010” (< > <), “011” (< > >), “110” (> > <), and “111” (> > >). Therefore, “111” represents that the USP gene and the two adjacent genes are in the same direction; while “010” represents that the USP gene is in a different transcription direction relative to the two adjacent genes. This representation could identify USP genes that have a different gene adjacency pattern from the majority of genomes from the same species. We designed visual analytics worksheets to integrate and group the USP genes according to gene transcription direction, location coordinates (start and stop positions), and pfam annotations of adjacent genes.

The protein function annotations using Cluster of Orthologous Groups (COGs) of proteins [49] and protein family (pfam) were obtained for adjacent genes. We used the “Conserved Neighborhood” tool of IMG to verify the gene transcription direction and the pfam annotation of the gene neighborhood group (010, 011, 110, or 111) assigned in the visual analytics worksheets. The web pages of a USP gene in BioCyc [50] were used to determine the presence of the USP gene in a putative operon.

### 2.5. Transcriptome and Interactome Evidence for Helicobacter pylori Universal Stress Protein

We searched scholarly literature databases such as Google Scholar and PubMed for studies on RNA-Sequencing (RNA-seq), transcriptome profiling, and protein–protein interaction maps of *Helicobacter pylori* that have supplementary data that included the universal stress protein HP0031. For RNA-seq studies, we prioritize studies with fold change and statistical significance data in the Supplementary Files.

## 3. Results

### 3.1. Universal Stress Protein Proteins Encoded by Helicobacteraceae Genomes

*Genomes in the Dataset*: Our query for genomes annotated as *Helicobacteraceae* in the Integrated Microbial Genome & Microbiome (IMG/M) system in January 2024 retrieved 1014 genomes from 999 unique strains. Nine *Helicobacter pylori* strains and one *Helicobacter suis* had two genome sequences in the dataset. One strain each of *Helicobacter canadensis*, *Helicobacter cinaedi*, and *Helicobacter heilmannii* also had two genomes. *Helicobacter bilis* ATCC 51630 had three genomes. Two strains (CG1_02_36_14 and CG2_30_36_10) had the annotation of unclassified *Helicobacteraceae* bacterium, obtained from a groundwater metagenome. We retained the two genomes in the data investigation to determine the features of the universal stress proteins and possibly infer the taxonomic association. The final dataset consisted of 1045 USP genes from 1011 genomes of *Helicobacter*, two genomes of *Wolinella*, and one genome of strain CG1_02_36_10. There were 47 *Helicobacter* (33 enterohepatic, one enterohepatic and gastric, and 13 gastric) with species name in the dataset (Table 1). In addition, 26 *Helicobacter* genomes with only strain identifiers (Supplementary Table S1) were grouped by anatomic niche and host organisms criteria as described in publications (such as Mannon et al. [51], O’Toole et al. [52], and Gilbert et al. [53]) as well as genome pages of the NCBI’s BioProject [54] and IMG/M system [32].

*Universal Stress Protein Gene Count per Genome*: In the dataset collected, there were 1045 predicted genes with the universal stress protein (USP) domain annotations from 1014 *Helicobacteraceae* genomes. In addition, 984 *Helicobacteraceae* genomes had one USP gene annotation while 29 *Helicobacteraceae* genomes had two annotations for the USP gene. The genome of *Helicobacter fennelliae* NCTC 13102 had three annotations for USP genes. In the case of *H. pylori*, 781 (99%) of the 785 genomes had annotations for one USP each. Three *H. pylori* strains with two USP genes per genome are UM163 (IMG/M Genome ID: 2639762898), UM171S (IMG/M Genome ID: 2648501688), and UM276S (IMG/M Genome ID: 2651869863). The genome sequencing status of all the *H. pylori* strains with two USPs is permanent drafts. Strain UM037 (IMG/M Genome ID: 2545824636) genome had three genes annotated with the Usp (PF00582) domain. Of the two strains of the unclassified *Helicobacteraceae* bacterium, strain CG2_30_36_10 had annotation for one USP gene. The total count of *H. pylori* USP sequences retrieved was 789.

*Lengths of Universal Stress Protein Sequences*: The 24 observed protein lengths in amino acids (aa) were 66, 68, 72, 78, 88, 89, 99, 104, 111, 121, 126, 136, 137, 138, 139, 140, 141, 146, 147, 273, 274, 278, 279, and 285 (Supplementary Table S2, Figure S1). Among the 1045 USP sequences, the top two frequent protein sequence lengths were 137 aa (785 sequences) and 138 (182 sequences). The *Helicobacteraceae* USP sequence lengths observed are in two categories, from 66 aa to 147 aa and 273 aa to 285 aa. One USP gene encoding 279 aa protein sequence was predicted from the genome of unclassified *Helicobacteraceae* strain CG2_30_36_10. The protein lengths less than 137 aa were from one 88 aa USP sequence from *H. bilis* ATCC 51630, and 14 USP genes from 12 *H. pylori* genomes: A45, CPY6081, Hp238, HPJP26, HPKX_438_AG0C1, UM137R, UM137S, UM163 (2 USP genes), UM171S, UM229R, UM276R, and UM276S (2 USP genes). The 137 aa sequences were predicted from only the gastric helicobacters species (*H. acinonychis*, *H. cetorum* and *H. pylori*). Additional description of the USP sequence length is available in the footer section Table S2.

### 3.2. Grouping of Helicobacteraceae Species by Patterns of Functional Sites in Universal Stress Protein Sequences

**Figure 1:**
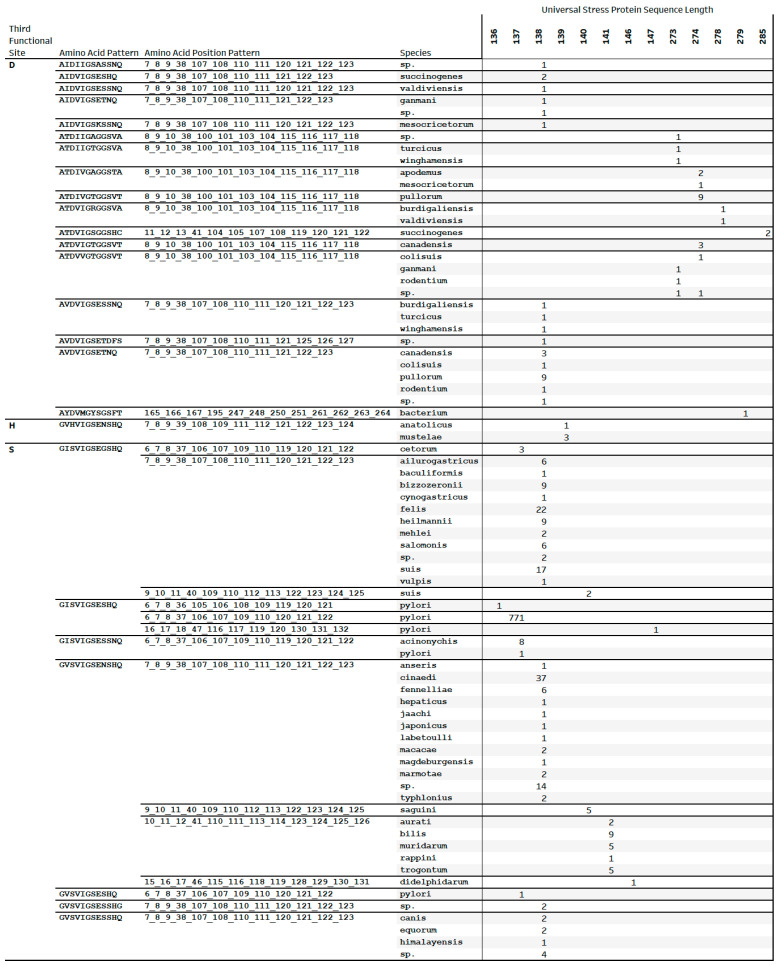
Grouping of species of *Helicobacter* and related taxa according to functional sites patterns in 1030 universal stress protein sequences defined by length. The three amino acids in the third position of the amino acid patterns, 25 amino acid patterns and 14 amino acid position patterns provided an approach to group 47 named species of *Helicobacter*, 26 species of *Helicobacter* without assigned species name (labeled sp.), *Wolinella succinogenes* and species of an unclassified related campylobacteria strain (labeled bacterium).

The NCBI Batch Web Conserved Domain (CD) Search Tool predicted functional sites for 1030 (98%) of the 1045 universal stress protein sequences submitted. The output of the search tool includes coordinates (e.g., G6, I7, S8, V36, I105, G106, S108, E109, S119, H120, and Q121), the complete size of the ligand (e.g., 12), and the mapped size (e.g., 11). We constructed an Amino Acid Pattern by combining the amino acids symbols (e.g., GISVIGSESHQ) and the Amino Acid Position Pattern by combining numeric position of the amino acid residue separated by an underscore (e.g., 6_7_8_36_105_106_108_109_119_120_121). In the third position of the USP functional sites patterns, there were three types of residues: aspartate (D), histidine (H) and serine (S). The residue in the third position was selected because changes in the amino acid affected ATP-binding [26,27]. The design of the grouping approach used the residue in the third position, which has the highest level of the grouping, followed by the amino acid pattern and the amino acid position pattern. We used the 25 amino acid patterns and the 14 amino acid position patterns to group 47 named species of *Helicobacter*, 26 species of *Helicobacter* without assigned names (labeled sp.), *Wolinella succinogenes* and species of an unclassified related campylobacteria strain (labeled bacterium in the species column) (Figure 1). The protein sequence length associated with the functional site patterns provided an additional feature in Figure 1 to facilitate interpretations and further investigations of taxa groups.

A use of our grouping approach is for identifying species with unique or identical functional sites patterns in *Helicobacteraceae* USP sequences. All the USPs with lengths 273 aa, 274 aa, 279 aa, and 285 aa have aspartate as residue in the third position. The 138 aa *Helicobacteraceae* USPs group into those with third position residue as aspartate (D) or serine (S). Among USP sequences with aspartate (D) in the third position of functional site patterns, the 273 aa of *H. turcicus* and *H. winghamensis* have an identical amino acid pattern of functional sites (ATDIIGTGGSVA). Other examples are (1) 273 aa USPs of *H. apodemus* and *H. mesocricetorum* with pattern ATDIVGAGGSTA; and (2) 278 aa USPs of *H. burdiagaliensis* and *H. valdiviensis* with pattern ATDVIGRGGSVA. The functional sites patterns of the 138 aa USP of *H. valdiviensis* is AIDVIGSESSNQ, which is distinct from AVDVIGSESSNQ from the 138 USPs of *H. burdigaliensis*, *H. turcicus*, and *H. winghamensis.* The only two 139 aa USPs with histidine (H) in the third position of functional site patterns were encoded in the genomes of *H. anatolicus* and three genomes of *H. mustelae*. The groupings by this approach are consistent with prior studies using other methods: *H. turcicus* and *H. winghamensis* [1]; *H. apodemus* and *H. mesocricetorum* [55]; *H. burdigaliensis* and *H. valdiviensis* [56]; and *H. anatolicus* and *H. mustelae* [1].

The 774 *H. pylori* USPs (136 aa, 137 aa, and 147 aa lengths) shared the same functional sites pattern of GISVIGSESHQ, where the residue in the third position is serine (S). A 137 aa USP encoded by *H. pylori* Aklavik86 and eight *H. acinonychis* shared functional sites coordinates with 12 positions (G6, I7, S8, V37, I106, G107, S109, E110, S119, S120, N121, and Q122). *H. pylori* Aklavik86 and *H. acinonychis* have shared genomic features [57]. Another use of the grouping approach is to predict the taxonomic or population identity of strains without a species name. *H. ganmani* USPs with sequence lengths 138 aa and 273 aa have aspartate in the third position and grouped with USPs of the same lengths, respectively, from *Helicobacter* sp. MIT 05-5294. Phylogenetic analysis clustered *H. ganmani* and strain MIT 05-5294 in the Enterohepatic *Helicobacter* Species clade 1 (EHS 1) [51]. Furthermore, the pattern GISVIGSEGSHQ is common to the 138 aa USP from 11 gastric helicobacters (*H. cetorum*, *H. ailurogstricus*, *H. baculiformis*, *H. bizzozeronii*, *H. cynogastricus*, *H. felis*, *H. heilmannii*, *H. mehlei*, *H. salmonis*, *H. suis*, and *H. vulpis*) as well as *Helicobacter* sp. L8 and *Helicobacter* sp. NHP19-012. According to entries in the Genomes OnLine Database [58], strain L8 and strain NHP19-012 are gastric helicobacters.

### 3.3. Similarity of Helicobacteraceae Universal Stress Proteins Sequences

**Figure 2:**
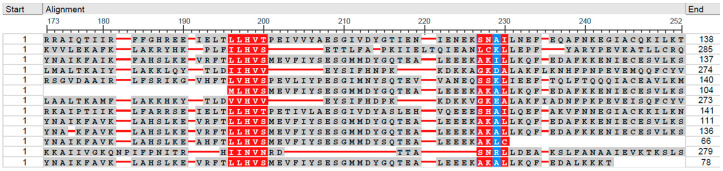
A section of multiple sequence alignment of 183 representative *Helicobacteraceae* universal stress proteins. The sequences, irrespective of their length, contained two sequence regions with highly conserved (red) positions and lower conserved positions (blue). The image was constructed using the NCBI Multiple Sequence Alignment Viewer.

We used the CD-HIT biological sequence clustering software [36] to cluster 1045 *Helicobacteraceae* USP sequences into 183 representative sequences (181 *Helicobacter*, 1 *Wolinella* and 1 unclassified *Helicobacteracae*). Consequently, our dataset of protein sequences contained 182 representative sequences from the taxonomically classified *Helicobacteracae* species. The initial set of protein sequences and the representative sequences are available as Supplementary Files S1 and S2, respectively. The multiple sequence alignment in fasta format, guide tree in Newick format, multiple sequence alignment view, and iTOL constructed phylogenetic tree for the 183 representative USP sequence are available as Supplementary Files S3, S4, S5 and S6, respectively. The clustering process assigned the 789 *Helicobacter pylori* USP sequences to 83 clusters with 54 clusters having one USP sequence. A view of the multiple sequence alignment with the NCBI Multiple Sequence Alignment Viewer, Version 1.25.0 identified stretches (without gaps) of highly conserved and lower conserved positions in the representative USP sequences (Figure 2). A consensus motif of LLHVS (Leucine–Leucine–Histidine–Valine–Serine) was identified from the alignment.

**Figure 3:**
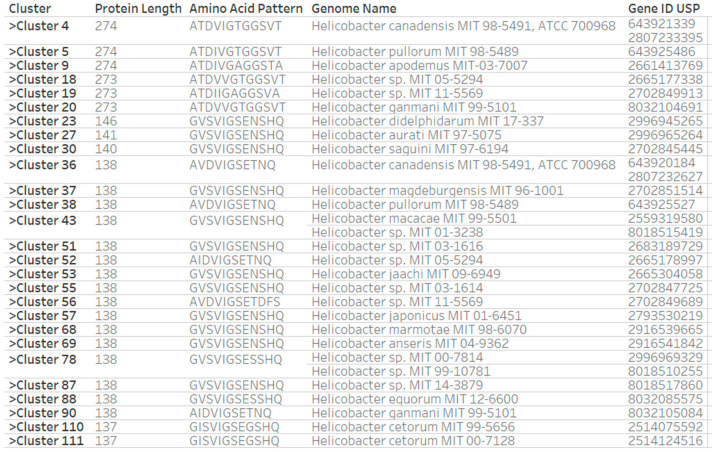
Clusters, sequence lengths, and amino acid patterns of functional sites for 31 universal stress proteins in 27 clusters of 24 strains of the *Helicobacter* MIT strain group. The visualization contains multiple findings. For example, the two genomes of *Helicobacter canadensis*, MIT 98-5491 and ATCC 700968, each encode two genes for universal stress proteins with amino acid lengths of 274 and 138. In addition, strain MIT 01-3238 in Cluster 43 could be a strain of *Helicobacter macacae*. Strains MIT 00-7814 and MIT 99-10781 in Cluster 78 could be strains of the same *Helicobacter* species.

The clustering of the 1045 USP sequences into 183 clusters also allowed us to determine the count of USP sequence clusters per group of strains (strain group). A strain group has strains, which share features such as a genome sequencing project or publication. For example, *H. pylori* strains with “SA” as the first two letters of strain name were isolated in South Africa [59]. We present in Table 2 the count of universal stress protein sequences and corresponding count of sequence clusters for selected strain groups with isolates from Gambia, South Africa, China, and Malaysia. In addition, we designed a worksheet in Tableau to determine unique and shared clusters among USP sequences in a strain group. The visual design of the worksheet had the following order of the fields: cluster, protein length, amino acid pattern (functional sites), genome name, and IMG Gene ID for USP gene (Figure 3 for MIT strain group and Supplementary Figure S2 for *H. pylori* GAM strain group). The MIT strain group, which is typically a *Helicobacter* species other than *H. pylori*, had 24 strains with strain name having the MIT-XX-XXXX suffix, 27 USP sequence clusters, and 31 USP sequences (Figure 3). We also observed that the IMG genome names of four MIT strains (05-5293, 11-8110, 15-1451, and 16-1353) did not include the suffix MIT. Furthermore, the full scientific name for strain 15-1451 is now *Helicobacter monodelphidis* MIT 15-1451 [60].

### 3.4. Predicted Protein Functions for Adjacent Genes of Universal Stress Protein Genes in Helicobacteraceae Genomes

We observed 36 predicted protein domains (pfam families) annotated for genes adjacent and in the same transcription direction with *Helicobacteraceae* USP genes. The encoded proteins have amino acid sequence lengths of 137 aa, 138 aa, 139 aa, 140 aa, 141 aa, 146 aa, 147 aa, 273 aa, 274 aa, 278 aa, and 285 aa (Supplementary Figure S3). Some proteins encoded by genes adjacent to USP genes had more than one protein domain. In addition, some adjacent genes in the same transcription direction did not have predicted protein functions. We used the four three-digit binary numbers (010, 011, 110, and 111) to analyze the gene adjacency pattern of each USP gene. Our visual analytics filtering procedure assigned 1009 *Helicobacteraceae* USP genes, located in the same IMG genome scaffold source as their adjacent genes, into one of four possible three-digit binary numbers representing gene transcription direction: 010 (11 USP genes), 110 (320 USP genes), 011 (599 USP genes), or 111 (119 USP genes) (Table S3). In Supplementary Figure S4, we present examples of *Helicobacteracae* USP genes in the four patterns of gene transcription direction obtained from the IMG/M system for three USP sequence lengths: 137 aa, 138 aa, and 273 aa. The genome of *Helicobacter winghamensis* ATCC BAA-430 encodes USP genes with gene transcription pattern 010 (138 aa) and 110 (273 aa). A complete figure with 23 USP example genes according to Table S3 is available as Supplementary File S7.

**Figure 4:**
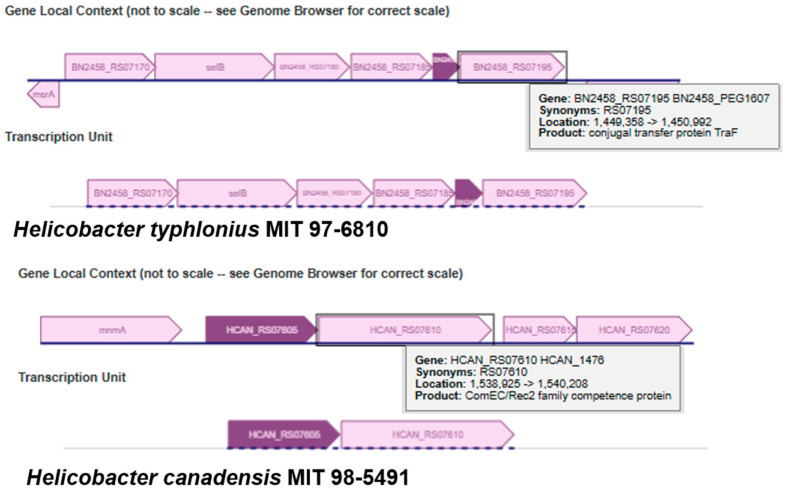
BioCyc diagrams of gene local context and transcription unit for genes for universal stress proteins adjacent to genes for proteins involved in DNA uptake. The universal stress protein (USP) gene image is darker than neighboring genes. The transcription unit indicates genes that could be in an operon. According to BioCyc Operons tab in the gene page, a dashed baseline indicates that there is no high-quality evidence to confirm the extent of this transcription unit. *Top diagram*: Gene encoding a 138 aa universal stress protein (USP) in the genome of *Helicobacter typhlonius* MIT 97-6810. *Bottom diagram*: Gene encoding a 274 aa USP in the genome of *Helicobacter canadensis* MIT 98-5491. Interactive diagrams can be accessed at the BioCyc website.

Among the USP genes of the *Helicobacter* genus, we prioritized six categories of biological processes for which the protein encoded by adjacent genes could function (Table 3). Genes encoding DNA uptake were adjacent to USP genes in the two categories of length (conjugal transfer protein TraF for < 200 aa and ComEC/Rec2 family competence protein for > 200 aa) (Figure 4). Five other biological process categories that we prioritized were (1) amino acid transport and metabolism; (2) coenzyme transport and metabolism; (3) membrane transport; (3) posttranslational modification, protein turnover, chaperones; (4) replication, recombination and repair; and (5) mediation of protein–protein interactions.

**Figure 5:**
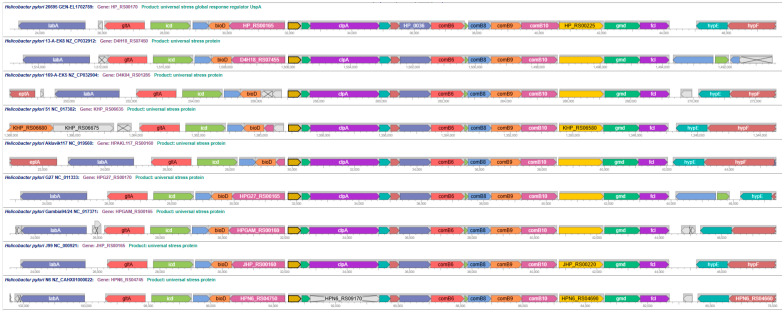
BioCyc multi-genome alignment of the genomic region of nine *Helicobacter pylori* genomes that includes the gene for universal stress protein. The *H. pylori* gene for universal stress protein (USP) image has diagonal lines. The predicted operon (grey filled rectangle) containing the USP gene in all strains except strain 26695 consists of 11 genes starting with the USP gene (HP0031) and ending with the DNA type IV secretion protein ComB10 (HP0041). Other predicted proteins in the operon are (1) HP0032: ATP-dependent Clp protease adapter protein ClpS); (2) HP0033: ATP-dependent Clp protease ATP-binding subunit ClpA; (3) HP0034: L-aspartate 1-decarboxylase; (4) HP0035: nucleoid-associated protein EbfC; (5) HP0036: PDZ domain protein; (6) HP0037: ComB6 competence protein; (7) ComB7; (8) HP0038: ComB8; (9) HP0040: ComB9; and (10): HP0041: ComB10. Interactive diagrams can be accessed at the BioCyc website.

We observed that the adjacent genes annotated with “posttranslational modification, protein turnover, chaperones” biological process category occurred exclusively in gastric helicobacters (Table 1 and Table 3). Furthermore, within the biological process category, there were three ATP-dependent protein families: (1) ATP-dependent Clp protease ATP-binding subunit ClpA [COG0542]; (2) ATP-dependent serine protease [COG1066]; and (3) ATP-dependent Clp protease adapter protein ClpS [COG2127] (Figure S4). The second protein family was encoded by an adjacent gene to the USP gene of *H. mustelae*. The helicobacters of three species (*H. acinonychis*, *H. cetorum* and *H. pylori*) had the gene for ClpS adjacent to the USP gene (Figure S5). An operon could contain one or both of the adjacent genes to a *Helicobacter* USP gene in our prioritized biological processes (Figure 5 and Figure S5). The number of genes predicted to be in the operon with the *H. pylori* USP gene was seven or 11 and included the genes for five DNA uptake competence proteins (ComB6, ComB7, ComB8, ComB9 and ComB10) (Figure 5). The gene for ComB7 is 117 bp and encodes a 35 aa protein. Finally, in the case of taxonomically unclassified *Helicobacteraceae* strain CG2_30_36_10, a search for gene neighborhood similarity in the IMG/M system identified similar regions in campylobacterial genomes of *Sulfurospirillum arcachonense* and *Arcobacter* species. The gene for sulfate transport is adjacent to the USP gene of strain CG2_30_36_10.

### 3.5. Transcriptome and Interactome Evidence for Helicobacter pylori Universal Stress Protein

We identified two relevant genome-wide gene expression datasets to identify biological processes that regulate the gene for *H. pylori* USP. The datasets were the National Center for Biotechnology Institute (NCBI) Gene Expression Omnibus (GEO) GSE227450 [67] and the European Bioinformatics Institute (EBI) ArrayExpress E-MTAB-13025 [68]. The publications associated with the studies included spreadsheet files with data on fold change and statistical significance for *H. pylori* genes including USP and its neighboring genes such as the ClpS gene.

**Figure 6:**
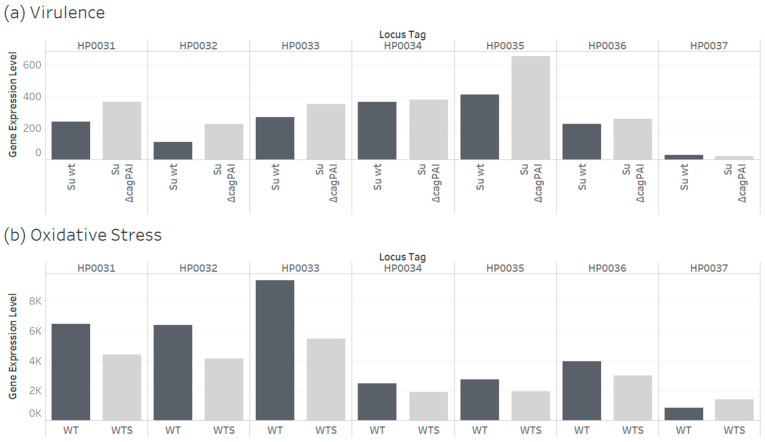
Evidence of expression of genes for *Helicobacter pylori* universal stress protein (HP0031) and ATP-dependent Clp protease adapter protein ClpS (HP0032) and other neighboring genes. (**a**) Gene expression of wild type strain (Su wt) and strain deficient in the cytotoxic-associated gene-pathogenicity island (Su ∆cagPAI). *Helicobacter pylori* Su isolate is from Northeast Africa, Sudan population [69]. (**b**) Gene expression of wild type strain (WT, incubated with 2% oxygen) and strain (WTS, incubated with 21% oxygen). (1) HP0033: ATP-dependent Clp protease ATP-binding subunit ClpA; (2) HP0034: L-aspartate 1-decarboxylase; (3) HP0035: nucleoid-associated protein EbfC; (4) HP0036: PDZ domain protein; (5) HP0037: ComB6 competence protein.

The GSE227450 dataset includes an RNA-sequencing (RNA-Seq) comparison of gene transcription between parental strain (Su wt) of *H. pylori* and a strain of *H. pylori* with a deficient cytotoxic-associated gene-pathogenicity island (Su ∆cagPAI). The ArrayExpress E-MTAB-13025 dataset includes an RNA-Seq comparison of gene transcription between *H. pylori* incubated in microaerophilic (optimal, WT) conditions and *H. pylori* incubated in aerobic (oxidative stress, WTS) conditions. In both experiments, the genes for USP (HP0031) and ClpS (HP0032) had statistically significant fold change values (Figure 6).

We identified a protein–protein interaction dataset of *H. pylori* that included interaction partners for universal stress protein, HP0031 [70]. Using a high-throughput strategy of the yeast two-hybrid assay, seven proteins encoded by genes with locus tags HP0006, HP0066, HP0281, HP1041, HP1513, and HP1567 interacted with *H. pylori* USP (HP0031) (Table 4).

### 3.6. Interactive Analytics Resources for Investigating Dataset on Helicobacteraceae Genomes and Universal Stress Protein Genes

We constructed a dataset of 1045 USP genes encoded by 1011 *Helicobacter* genomes, two *Wolinella*, and an unclassified *Helicobacteraceae* bacterium CG2_30_36-10. The dataset has 46 data columns that are in five categories, and the count of data columns is Gene Annotation (18), Gene Adjacency (7), Genome Annotation (9), Phylogeography of *H. pylori* (5), and Protein Sequence (7). This dataset and description of each data column is available as Supplementary File S8. We designed the dataset and web resource for researchers to use the USP sequence clusters and gene adjacency distances to group *H. pylori* populations according to geographic or *H. pylori* population factors. A subset of 773 *H. pylori* USPs were identified to be adjacent to the gene for ClpS and have a protein length of 137 aa. The sequence analysis and gene adjacency procedures respectively clustered the USPs by sequence similarity to 78 clusters and grouped the USPs by the 24 distances between the genes for ClpS and USP. A cluster of 474 USP sequences (Cluster 22) was the largest cluster, followed by Cluster 102 with 119 USP sequences.

Combining the two grouping procedures with the distance pattern as the first level and sequence similarity as the second level revealed groups with multiple *H. pylori* populations or from a specific *H. pylori* population (examples in Table 5). The distances between the USP gene and adjacent genes did not change in experiments that determined changes such as mutation to genomic regions of input and output strains, as observed with BCM-300 and HE labeled strains as well as J166 and J166ouput strains.

**Figure 7:**
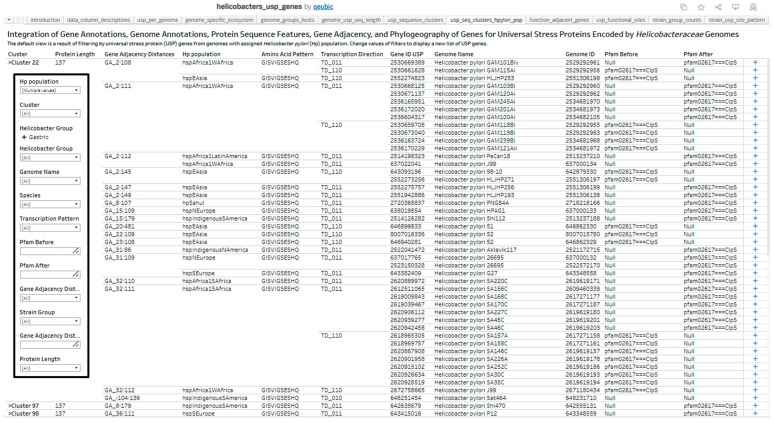
A section of an image from the web resource for interacting with the dataset composed of values for annotations of universal stress protein sequences encoded by *Helicobacter pylori* genomes with assigned population. The categories of annotations in the visual are (1) Gene Annotations (Gene ID USP); Genome Annotations (Genome ID, Genome Name, Helicobacter Group, Species, and Strain Group); Protein Sequence Features (amino Acid Pattern, Cluster, and Protein Length), Gene Adjacency (Gene Adjacency Distances, Pfam Before, Pfam After, and Transcription Direction); and Phylogeography of *H. pylori* isolates (Hp population). The insert rectangle shows filters that can be modified to change the list of genes displayed. The population ancestry was obtained from The *Helicobacter pylori* Genome Project [38]. We obtained the additional annotations from the Integrated Microbial Genomes/Microbiomes (IMG/M) system or derived them with bioinformatics/visual analytics procedures.

We developed an online (Tableau Public) web resource consisting of interactive analytics worksheets and dashboards that allow for further data investigations and research on several aspects of *Helicobacteraceae* universal stress proteins. The website address to the resource is provided in the Data Availability section. The online resource includes interactive versions of figures in this report. In addition, static images of all the figures in this report are also available for viewing in the web resource. We present an example of interactive analytics worksheet with filters, named “usp_seq_clusters_hyplori_pop” (Figure 7). This interactive worksheet displays values of variables for 84 USP genes by categories of gene annotation, genome annotation, protein sequence features, gene adjacency, and phylogeography of genes for universal stress proteins encoded by *H. pylori* genomes.

The visual revealed the subgroups within clusters of USP genes according to the gene adjacency distances (base pairs between the USP gene and each adjacent gene), population ancestry of *H. pylori* isolate, pattern of amino acid functional sites, and the binary number of the transcription direction of the USP gene and adjacent genes. Among isolates with assigned population ancestry, the 11 distance (bp) values observed between the USP gene and the gene for ClpS (ATP-dependent caseinolytic protease (Clp) adaptor protein) were 2, 7, 8, 15, 20, 22, 23, 31, 32, 36, and 53. The grouping by gene adjacency distances revealed USP sequences from different *H. pylori* populations that share values of gene adjacency distances. For example, hspAfrica1WAfrica (GAM101Bv and GAM115Ai) and hspEAsia (HLJHP253) have the 2-bp between the USP and ClpS genes. We also observed findings unique to strain groups. All the 45 GAM (isolates from The Gambia, West Africa) genomes have the 2-bp distance. Among 97 South Africa (SA) *H. pylori* genomes, four base pair distances (2, 15, 32, and 44) occurred between the genes for USP and ClpS. The gene adjacency (−104:136) and transcription direction pattern (010) for strain Sat464 (*H. pylori* population: hspIndigenousSAmerica) are atypical compared with other *H. pylori* strains because in the IMG/M genome annotation there is an adjacent upstream gene in opposite genome strand (IMG/M Gene ID: 648251455) to the USP Gene (IMG/M Gene ID: 648251454). The ClpS gene is on the same strand as the USP gene and has the IMG Gene ID of 648251456.

## 4. Discussion

We have conducted an in-depth data investigation of universal stress proteins (USPs) encoded by genomes of *Helicobacter pylori* and related *Helicobacteraceae* bacteria. To facilitate reuse of data, research, and applications, we produced interactive analytics resources of a dataset composed of values for variables on *Helicobacteraceae* universal stress protein, including phylogeography of *H. pylori* strains, protein sequence features, and gene neighborhood. We combined bioinformatics and visual analytics approaches to investigate 1045 universal stress protein sequences encoded in 1014 *Helicobacteraceae* genomes, including 785 *Helicobacter pylori* genomes. The study generated a representative set of 183 universal stress protein (USP) sequences consisting of 180 *Helicobacter* sequences, two *Wolinella succinogenes* sequences, and a sequence from a related campylobacteria, which could serve as an outgroup sequence. This non-redundant collection of 183 USP sequences and the initial 1045 USP sequences are available as Supplementary Files. These sequences can be used for structural, evolutionary, and functional studies of *Helicobacteraceae* USPs. Such studies have helped to deepen the knowledge of universal stress proteins in different organisms including pathogens [17,26,30,79,80,81].

Genomic context searches and analysis identified USP genes of gastric and enterohepatic helicobacters that are in adjacency or operonic arrangements with genes for oxidative stress response proteins (ATP-dependent proteases: ClpS and ClpA); and DNA uptake proteins (natural competence for transformation proteins: ComB6, ComB7, ComB8, ComB9, ComB10, ComBE, and conjugative transfer signal peptidase: TraF). The ComB6, ComB7, ComB9, and ComB10 are necessary for the first step of DNA uptake in *H. pylori* [68,82]. In *Escherichia coli*, the genes for ClpS and ClpA occur as an operon and are induced by environmental hydrogen peroxide that can diffuse into the cell [83]. Furthermore, ClpS interacts with ClpA in an ATP-dependent manner [84]. The *E. coli* protein complex, ClpSAP (ClpS, ClpA, and ClpP) balances the protection against DNA damage (due to hydroxyl radicals produced from the reaction of hydrogen peroxide with ferrous cation) and the availability of iron required for iron-dependent enzymes for DNA repair pathways and cell viability [83]. A mechanism for the development of gastric cancer is that severe oxidative stress in the gastric mucosa in response to *H. pylori* colonization leads to increased protein expression of virulence factors (CagA, VacA, and AlpA) that cause repeated inflammation of gastric epithelial cells and increased production of antioxidant enzymes (AhpC, KatA, and HtrA) to protect *H. pylori* [85]. A search with the chromosomal cassette search tool in the IMG/M database for genomes that contain genes for USP, ClpS, and ClpA in same chromosomal cassette revealed that the genomic context where gene for USP is adjacent downstream to gene for ClpS is unique to the gastric helicobacters (*H. acinonychis*, *H. cetorum*, and *H. pylori*). Experimental studies will help verify genomic context and elucidate functions of the gene neighborhood of *Helicobacteraceae* USP genes in oxidative stress response and virulence.

Our analysis of datasets from transcript profiling experiments verified that *H. pylori* USP and ClpS genes are expressed in wild-type strains and are regulated by the cagPAI region and oxidative stress condition [67,68] (Figure 6). The cagPAI genomic region encodes a type IV secretion system (T4SS), which is a virulence factor that is involved in carcinogenesis [86]. Furthermore, we provided protein–protein interaction evidence for the interaction of *H. pylori* USP with seven proteins, including (1) DNA translocase FtsK with ATP-binding motif, and (2) flagellar biosynthesis protein FlhA [70]. A DNA recombination/repair protein (RecA)/FtsK-dependent regulatory pathway regulates the universal stress protein A of *Escherichia coli* [87]. The *H. pylori* FlhA is part of the type III secretion system of the flagellar basal body [88]. Future studies could determine the genes that regulate USP and ClpS genes. These studies could provide evidence for gene networks that involve both genes in *H. pylori* and related gastric helicobacters, such as *H. acinonychis* and *H. cetorum*.

We used the functional site patterns of 1030 universal stress proteins predicted with NCBI Conserved Domain search to group *Helicobacter* species, *Wolinella succinogenes* and an unclassified related bacterium (Figure 1). The predictions revealed the presence of 11 and 12 functional sites among 25 amino acid patterns and 14 amino acid position patterns. There was independent evidence, including phylogenetic analysis, to support the use of the universal stress protein-based grouping for assigning taxonomic or population annotations to genomes. The *Helicobacteraceae* USPs with protein sequence lengths 273 aa, 274 aa, 278 aa, and 285 aa have functional sites patterns in which the three typical glycine residues are present with 10 residues between the second and third glycine residues in the known ATP-binding motif. Thus, the predicted ATP-binding motif in the *Helicobacter* and *Wolinella* USPs is G2XG10GXS compared with the typical G2XG9XG(S/T) of the ATP-binding *Methanocaldococcus jannaschii* USP [17,18]. These functional site patterns matched with protein lengths can provide a guide for planning investigations on interactions of USPs of *Helicobacter* and *Wolinella* with ATP and other molecules. Publications on *Helicobacter* genes and proteins (e.g., [89,90]) as well as USPs of other bacteria (e.g., [26,27]) can provide techniques for investigating the interaction with ATP and other molecules by *Helicobacteraceae* universal stress proteins.

The 1045 USP sequences from 1014 genomes of *Helicobacter*, *Wolinella*, and an unclassified strain revealed a remarkable diversity in protein lengths (Table S2 and Figure S1) and functional site patterns (Figure 1). The predominant USP sequence lengths of 137 aa and 138 aa, observed in gastric and non-pylori *Helicobacter* species, respectively, suggest potential functional specialization. This distinction may reflect adaptations to different host environments and stress conditions encountered by various *Helicobacter* species. Additionally, the identification of highly conserved and lower conserved regions in the multiple sequence alignment of representative USP sequences, along with the consensus motif of LLHVS (Figure 2), suggests functional importance and potential evolutionary constraints on these proteins. These conserved regions could serve as targets for future studies on USP function and as potential markers for diagnostic or therapeutic interventions [91,92]. The 15 USP genes encoding protein lengths of less than 137 aa could be further investigated for accuracy of the gene predictions. Nine of these USP genes are encoded in genomes of *H. pylori* strains isolated in Malaysia (UM137R, UM137S, UM163, UM171S, UM229R, UM276R, and UM276S). For strain UM163, the IMG/M system predicted two USP genes that code for 99 aa (2641177340) and 121 aa (2641177784). Strain UM163 is classified as a good biofilm former [93]. Since universal stress proteins can function in biofilm formation [30,31], the list of 31 *H. pylori* strains [93] of the UM strain group that are classified as poor, moderate, and good biofilm formers, could aid investigations on *Helicobacter* USP functions in biofilm formation.

The functional annotations and transcription direction of genes adjacent to USP genes have provided valuable insights into potential functional associations and operon arrangements. Noteworthy is the finding that genes involved in posttranslational modification, protein turnover, and chaperone functions are adjacent to USP genes exclusively in gastric helicobacters. The USPs in these species may function in protein synthesis quality control and stress response mechanisms specific to the gastric environment [94]. Krüger et al. proposed that the import of huge amounts of non-homologous DNA might establish a dilution effect through a reservoir of oxidizable nucleotides, thereby protecting *H. pylori*’s cytoplasmic chromosomal DNA against oxidative stress, in particular during host immune response [95]. Some bacteria USPs contribute to oxidative stress defense [20,21]. The *H. pylori* DNA, proteins and lipids are targets for host oxidative stress [96,97]. The adjacency of DNA-uptake genes to *Helicobacter* USP genes indicates that further research is needed to determine the roles of *Helicobacter* USPs in oxidative stress defense.

The analysis of *H. pylori* strains from different geographical regions, such as Gambia, South Africa, China, and Malaysia, has revealed strain-specific variations in USP sequences and genomic contexts. These differences may reflect adaptations to diverse host populations and environmental conditions such as exposure to antibiotics. The observed variations in gene adjacency distances between USP and ClpS genes among different *H. pylori* populations highlight the dynamic nature of genome organization in this species and suggest potential functional implications of these arrangements [46]. We have developed datasets and an interactive web resource (Figure 7) to support further research on the effects of USP genomic context and protein sequence features on *H. pylori* populations. Thus, datasets on antibiotic resistance [62] and efficiency of biofilm formation [93] could be integrated into future analysis.

The ClpS is an adaptor that binds N-terminal residues (tyrosine, phenylalanine, tryptophan, and leucine) and delivers attached substrates to the AAA + ClpAP protease for degradation [98,99]. In *H. pylori* genomes, the gene for ClpS is adjacent to the gene for the ClpA chaperone of the ClpAP complex (Figure 6 and Figure S4). These figures show that the two proteins involved in stress response are encoded in the same operon as the USP. The *H. pylori* strains with defective ClpA or ClpP (proteolytic component) of ClpAP were sensitive to some antimicrobials [100]. In *Campylobacter jejuni*, a closely related bacterium to *H. pylori*, ClpP is required for natural competence and DNA uptake [101]. The ClpS of *H. pylori* has unusual amino acid substitutions that may alter the binding specificity [102]. Since the gene for ClpS is not adjacent to the USP gene in non-*H. pylori* gastric helicobacters and in some *H. pylori* strains of West African origin [103], our findings revealed a need for research on the genomic context of the USP gene of *H. pylori* isolates, including implications on gastric cancer prevalence rates. A 2023 publication of a Kenyan eight-year study on the pattern and trends of *Helicobacter pylori* genotypes in gastric cancer concluded that “further assessment of the specific genes encoded by *H. pylori* isolates, in chronically infected persons, can aid in stratifying those at increased risk for development of gastric adenocarcinoma [104]”. Therefore, we recommend research studies on features of USP, ClpS and ClpA genes or proteins of *H. pylori* isolates from different clinical presentations of *H. pylori* infection in populations. In Africa, such studies on the universal stress proteins of *Helicobacteraceae* isolated in Africa would contribute data, information, and evidence for knowledge [105] to address identified research priorities [106,107].

The results of genomic context of USP genes revealed that the unclassified strain CG2_30_36_10, whose genome was from metagenomics studies of groundwater (Freshwater microbial communities from Crystal Geyser, Utah, USA, has USP gene adjacency to sulfate transport similar to *Arcobacter*, *Sulfurimonas*, and *Sulfurospirillum* genera. Within the phylum *Campylobacterota*, strains in the *Arcobacter*, *Sulfurimonas*, *Sulfurospirillum* genera inhabit extreme sulfidic habitats [108,109]. This observation suggests that genomic context analysis can contribute to the taxonomic identification of genomes from metagenomics studies.

This data investigation project relies on the integration of datasets from multiple sources. Consequently, changes to datasets or sources of data can influence the findings. To mitigate this limitation, we have included Supplementary Files and a web resource (Figure 7) as part of this report. In addition, where possible, we confirmed content of our dataset with published findings. For example, our dataset contains the same 97 genomes with the SA (South Africa) strain prefix, which is consistent with the genome count reported in the original publication [59]. In addition, the gene prediction algorithms could influence the prediction of genes in a genome. Thus, to confirm the presence or absence of genes and proteins, experimental assays may be needed, or multiple sources of gene annotations from microbial web portals, such as IMG/M, BioCyc, and BV-BRC (Bacteria and Viral–Bioinformatics Resource Center) [32,48,50,110].

## 5. Conclusions

There has been limited knowledge on the functions of the universal stress proteins encoded in the genomes of the *Helicobacteraceae* bacterial family, which consisted of *Helicobacter* and *Wolinella* genera. We have reported a more comprehensive data investigation of *Helicobacteraceae* universal stress proteins. New insights into their functions were obtained through a combination of bioinformatics and visual analytics approaches. Our findings suggest several promising avenues and applications in the field of *Helicobacter* biology and pathogenesis. These include further investigations on the functional characterization of *Helicobacteraceae* universal stress proteins from genomic context and protein sequence perspectives. Such studies would elucidate their specific roles in stress response and adaptation mechanisms. In addition, the potential operonic association of *H. pylori* USP in oxidative stress response and DNA uptake warrants further research. Finally, findings contribute to the knowledge of *Helicobacter* biology and may inform future strategies for managing *H. pylori* infections and associated diseases, including gastric cancer.

## Figures and Tables

**Table 1:** Grouping of *Helicobacter* genomes with species name encoding universal stress proteins by anatomic niche and host organism.

*Helicobacter* Group by Anatomic Niche	Host Organism ^1^	*Helicobacter* Species
Enterohepatic	Bird	*H. anatolicus*, *H. anseris*, *H. pullorum*, *H. valdiviensis*
	Dog	*H. canis*
	Hamster	*H. aurati*, *H. mesocricetorum*
	Horse	*H. equorum*
	Human	*H. bilis*, *H. burdigaliensis*, *H. canadensis*, *H. cinaedi*, *H. fennelliae*, *H. labetoulli*, *H. rappini*, *H. trogontum*, *H. winghamensis*
	Marmot	*H. marmotae*, *H. himalayensis*
	Marmoset	*H. jaachi*
	Monkey	*H. macacae*, *H. saguini*
	Mouse	*H. apodemus*, *H. ganmani*, *H. hepaticus*, *H. japonicas*, *H. magdeburgensis, H. rodentium*, *H. typhlonius*
	Opossum	*H. didelphidarum*
	Pig	*H. colisuis*
	Rat	*H. muridarum*
	Squirrel	*H. turcicus*
Enterohepatic and Gastric	Ferret	*H. mustelae*
Gastric	Cat	*H. ailurogastricus*, *H. baculiformis*, *H. felis*, *H. heilmannii*
	Cheetah, Lion, Tiger	*H. acinonychis*
	Dog	*H. bizzozeronii*, *H. cynogastricus*, *H. salomonis*
	Dolphin	*H. cetorum*
	Fox	*H. mehlei*, *H. vulpis*
	Human	*H. pylori*
	Pig	*H. suis*

^1^ Some *Helicobacter* species have been associated with more than one host organism.

**Table 2:** Counts of universal stress protein sequences and unique sequence clusters for selected strain groups of *Helicobacter pylori*.

Strain Group ^1^	Country of Isolation	Count of Strains	Count of Universal Stress Protein Sequences	Count of Sequence Clusters	Reference for Strain Group
GAM	Gambia	45	45	4	
SA	South Africa	97	97	11	[59,61]
Hpfe	China	95	95	21	[62]
UM	Malaysia	61	68	17	[63,64]
CPY	Japan	9	9	5	[65]
PUNO	Peru	9	9	4	[66]
Hp	USA	74	74	11	[65]

^1^ Prefix associated with the strain designation.

**Table 3:** Biological process categories of predicted proteins encoded by adjacent genes in the same transcription direction as universal stress protein (USP) genes of *Helicobacter* species.

Universal Stress Protein Sequence Length Category	Biological Process Category of Predicted Function of Adjacent Gene to Universal Stress Protein Gene	*Helicobacter* Species
<200 aa	Energy production and conversion	*H. aurati*, *H. muridarum*
	Amino acid transport and metabolism	*H. bilis*, *H. canadensis*, *H. colisuis*, *H. pullorum*, *H. macacae*
	Coenzyme transport and metabolism	*H. anatolicus*, *H. cinaedi*, *H. equorum*, *H. hepaticus*, *H. himalayensis*, *H. jaachi*, *H. japonicus*, *H. labetoulli*, *H. magdeburgensis*, *H. marmotae*, *H. mesocricetorum*, *H. typhlonius*, *H. valdiviensis*
	Replication, recombination, and repair	*H. anatolicus*, *H. mustelae*
	Posttranslational modification, protein turnover, and chaperones	*H. acinonychis*, *H. ailurogastricus*, *H. baculiformis*, *H. bizzozeronii*, *H. cetorum*, *H. cynogastricus*, *H. felis*, *H. heilmannii*, *H. mehlei*, *H. mustelae*, *H. pylori*, *H. salomonis*, *H. suis*, *H. vulpis*
	DNA Uptake	*H. canis*, *H. cinaedi*, *H. fennelliae*, *H. hepaticus*, *H. jaachi*, *H. japonicus*, *H. labetoulli*, *macacae*, *H. magdeburgensis*, *H. marmotae*, *H. typhlonius*
	Mediation of protein–protein interactions	*H. ganmani*
>200 aa	DNA Uptake	*H. canadensis*, *H. colisuis*, *H. ganmani*, *H. pullorum*, *H. rodentium*, *H. turcicus*, *H. valdiviensis*, *H. winghamensis*
	Membrane transport	*H. ganmani*, *H. rodentium*

**Table 4:** Proteins with evidence of interaction with *Helicobacter pylori* universal stress protein (HP0031).

Locus Tag *	Gene Symbol	Protein Name	Function Category
HP0006	*panC*	Pantoate—beta-alanine ligase	Coenzyme transport and metabolism
HP0066		DNA translocase FtsK	Cell cycle control, cell division, and chromosome partitioning
HP0281	*tgt*	tRNA guanosine(34) transglycosylase Tgt	Nucleotide transport and metabolism
HP1041	*flhA*	Flagellar biosynthesis protein FlhA	Cell motility
HP1513		L-seryl-tRNA(Sec) selenium transferase	Translation, ribosomal structure, and biogenesis
HP1567		Ribosome biogenesis GTP-binding protein YihA/YsxC	Cell cycle control, cell division, and chromosome partitioning

* Data source is Supplementary Data from protein–protein interaction map of *H. pylori* [70].

**Table 5:** Groupings of *Helicobacter pylori* populations by combining gene adjacency distances and sequence similarity of amino acid sequences for *H. pylori* universal stress protein.

Gene Adjacency Distances *	Sequence Cluster	*H. pylori* Population or County and Strains in Sequence Cluster	Notes and References
8:171	Cluster 22	hpEurope: BCM-300, HE101/09, HE132/09, HE136/09, HE141/09, HE142/09, HE143/09, HE147/09, HE170/09, HE171/09, HE178/09	HE labeled strains are re-isolates from human volunteers experimentally challenged with strain BCM-300 [71].
10:111	Cluster 22	hpEurope: J166, J166output_1moA, J166output_1moB, J166output_1moC, J166output_1wkA, J166output_1wkB, J166output_1wkC, J166output_2moA, J166output_2moB, J166output_2moC, J166output_6moA, J166output_6moB, J166output_6moC	J166 output isolates are from time points after experimental infection of a rhesus macaque with J166 [72].
31:109	Cluster 22	hpEurope: 26695, 26695 dRdM2addM2, 26695-1, 26695-1CH, 26695-1CL, 26695-1MET, 26695-dR, 26695-dRdM1dM2, 26695-dRdM2, dRdM1, G27, HP2RS, Rif1, Rif2	The strains are derived from or closely related to strain 26695 [73]. HP2RS indicates *H. pylori* 26695-related sequence [74]. G27 and 26695 have the same fundamental structure of the lipopolysaccharide of the outer membrane protein, a key factor for colonization and persistence in gastric niche [75].
31:109	Cluster 102	Australia: JCM 12093, CCUG 17874 hpEAsia: Hpfe0001	JCM 12093, CCUG 17874, NCTC 11637, and ATCC 43504 are equivalent strains [58]. CCUG 17874 is a CagA and VacA producing strain [76,77]. There is genomic sequence evidence that Hpfe0001 is closely related to CCUG 17874 and ATCC 43504 [78].
44:126	Cluster 103	hpAfrica2: SA47A, SA47C	*H. pylori* isolates from atrium (SA47A) and corpus (SA47C) stomach regions of same individual [59,61].

* First number is the distance (base pairs) between the ClpS gene and the USP gene. The second number is the distance between the USP gene and the other adjacent gene.

## Data Availability

Genomes of *Helicobacteraceae* can be retrieved from the IMG/M website: https://img.jgi.doe.gov/cgi-bin/m/main.cgi?section=TaxonList&page=lineageMicrobes&domain=Bacteria&family=Helicobacteraceae (accessed on 8 March 2025). The dataset on genes annotated with the pfam domain (pfam00582) for universal stress proteins can be retrieved from IMG/M https://img.jgi.doe.gov (accessed on 8 March 2025). The web resource with interactive analytics resources is available at Tableau Public by searching for helicobacter_usp_genes or at this web link: https://public.tableau.com/app/profile/qeubic/viz/helicobacters_usp_genes/introduction (accessed on 8 March 2025).

## Supplementary Materials

The following supporting information can be downloaded at: https://www.mdpi.com/article/10.3390/pathogens14030275/s1, File S1: The 1045 *Helicobacteraceae* universal stress protein sequences in FASTA format collected from IMG/M-ER; File S2: The 183 representative *Helicobacteraceae* sequences; File S3: Multiple sequence alignment file for 183 representative universal stress protein sequences in fasta format; File S4: Guide tree for 183 representative universal stress protein sequences in Newick format; File S5: Multiple sequence alignment view of 183 representative universal stress protein sequences with NCBI Multiple Sequence Alignment Viewer; File S6: iTOL constructed phylogenetic tree of the 183 representative universal stress protein sequences; File S7: Figure of gene transcription pattern of adjacent genes of 23 Helicobacteraceae universal stress protein genes; File S8: Two worksheets: (1) Descriptions of 46 data columns (variables) in dataset, and (2) Dataset of values of 46 data columns (variables) for 1045 Helicobacteraceae USPs. The following supplementary tables and figures are available in File S9. Table S1. *Helicobacter* genomes without species name encoding universal stress proteins grouped by anatomic niche and host organism. Table S2. Lengths of universal stress proteins encoded by *Helicobacteraceae* genomes. Table S3. Counts of *Helicobacteracae* universal stress protein sequence lengths according to gene transcription direction binary patterns. Figure S1. Protein sequence lengths of 1030 universal stress proteins encoded in 1002 genomes from species of *Helicobacter* and *Wolinella* as well as an unclassified *Helicobacteraceae* bacterium. Figure S2. Clusters, sequence lengths, and amino acid pattern of functional sites for 45 universal stress proteins in four clusters of 45 strains of the *Helicobacter pylori* GAM strain group (Isolates from The Gambia, West Africa). Figure S3. Predicted protein domains encoded by genes adjacent and in same transcription direction to genes for universal stress proteins in *Helicobacter* and *Wolinella* genomes. Figure S4. The gene transcription direction patterns (010, 011, 110, and 111) for three protein sequence lengths (137 aa, 138 aa, and 273 aa) of selected *Helicobacter* universal stress proteins (USP). Figure S5. Integration of gene neighborhood diagrams of universal stress proteins genes (with red box) in genomes of selected helicobacters with an adjacent gene for ATP-dependent protease. References [32,111] are cited in the supplementary materials.

## Abbreviations

The following abbreviations are used in this manuscript:

AAA+	ATPases associated with various cellular activities
AlpA	Adherence-associated lipoprotein A
AhpC	Alkyl hydroperoxide reductase
ATP	Adenosine Tri-Phosphate
CagA	Cytotoxin-associated gene A
DNA	Deoxyribonucleic acid
HtrA	High temperature requirement A protease
KatA	Catalase
NCBI	National Center for Biotechnology Information
VacA	Vacuolating cytotoxin A
USP	Universal Stress Protein
