# Supplementary material for: Insights into Functions of Universal Stress Proteins Encoded by Genomes of Gastric Cancer Pathogen Helicobacter pylori and Related Bacteria
Source: Pathogens. 2025 Mar 13;14(3):275. doi: 10.3390/pathogens14030275 (PMC11944479; doi:10.3390/pathogens14030275)
Supplement: Supplementary file 1 [file pathogens-14-00275-s001.zip › Supplementary-File-S5.pdf]

NCBI Multiple Sequence Alignment Viewer, Version 1.25.0

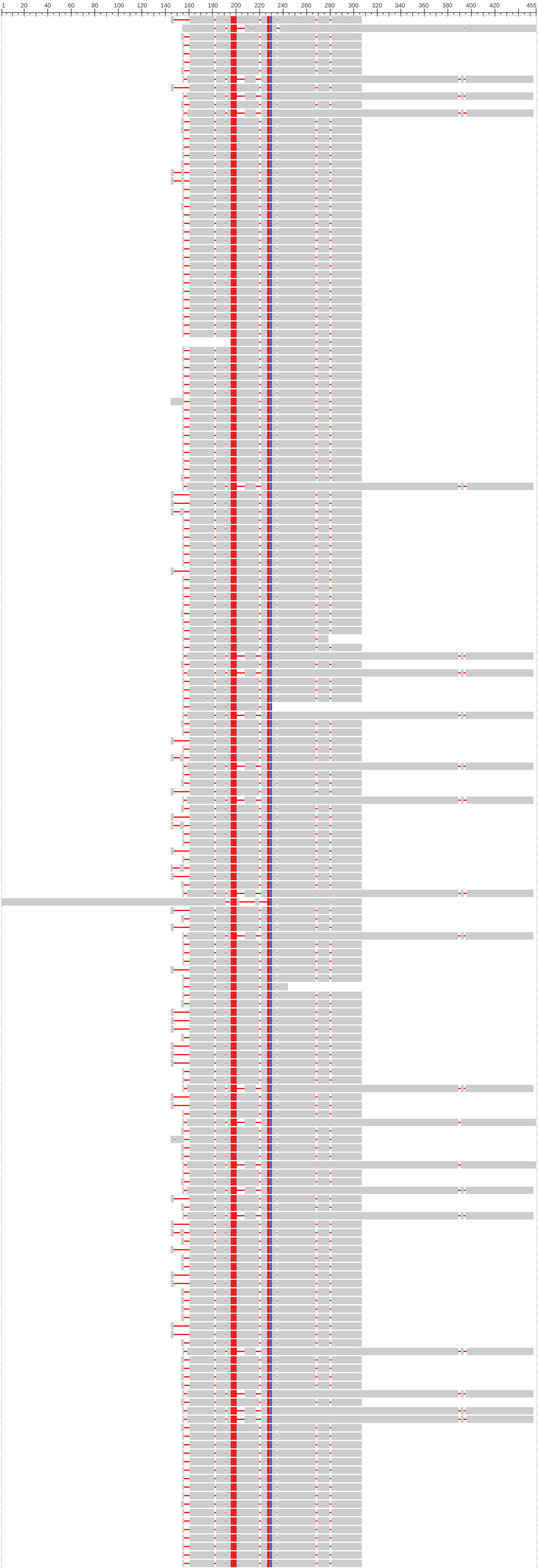

Sequence ID		Start	End	Organism
Query_10001	(+)	1	138	
Query_10002	(+)	1	285	
Query_10003	(+)	1	138	
Query_10004	(+)	1	137	
Query_10005	(+)	1	137	
Query_10006	(+)	1	137	
Query_10007	(+)	1	138	
Query_10008	(+)	1	274	
Query_10009	(+)	1	138	
Query_10010	(+)	1	274	
Query_10011	(+)	1	138	
Query_10012	(+)	1	273	
Query_10013	(+)	1	138	
Query_10014	(+)	1	139	
Query_10015	(+)	1	137	
Query_10016	(+)	1	137	
Query_10017	(+)	1	137	
Query_10018	(+)	1	138	
Query_10019	(+)	1	140	
Query_10020	(+)	1	140	
Query_10021	(+)	1	137	
Query_10022	(+)	1	137	
Query_10023	(+)	1	138	
Query_10024	(+)	1	137	
Query_10025	(+)	1	137	
Query_10026	(+)	1	137	
Query_10027	(+)	1	137	
Query_10028	(+)	1	137	
Query_10029	(+)	1	137	
Query_10030	(+)	1	137	
Query_10031	(+)	1	137	
Query_10032	(+)	1	137	
Query_10033	(+)	1	137	
Query_10034	(+)	1	137	
Query_10035	(+)	1	137	
Query_10036	(+)	1	137	
Query_10037	(+)	1	137	
Query_10038	(+)	1	137	
Query_10039	(+)	1	104	
Query_10040	(+)	1	137	
Query_10041	(+)	1	137	
Query_10042	(+)	1	137	
Query_10043	(+)	1	137	
Query_10044	(+)	1	137	
Query_10045	(+)	1	137	
Query_10046	(+)	1	147	
Query_10047	(+)	1	137	
Query_10048	(+)	1	137	
Query_10049	(+)	1	137	
Query_10050	(+)	1	137	
Query_10051	(+)	1	137	
Query_10052	(+)	1	137	
Query_10053	(+)	1	137	
Query_10054	(+)	1	137	
Query_10055	(+)	1	138	
Query_10056	(+)	1	273	
Query_10057	(+)	1	138	
Query_10058	(+)	1	138	
Query_10059	(+)	1	141	
Query_10060	(+)	1	137	
Query_10061	(+)	1	137	
Query_10062	(+)	1	137	
Query_10063	(+)	1	137	
Query_10064	(+)	1	137	
Query_10065	(+)	1	137	
Query_10066	(+)	1	138	
Query_10067	(+)	1	137	
Query_10068	(+)	1	137	
Query_10069	(+)	1	137	
Query_10070	(+)	1	137	
Query_10071	(+)	1	138	
Query_10072	(+)	1	137	
Query_10073	(+)	1	137	
Query_10074	(+)	1	111	
Query_10075	(+)	1	137	
Query_10076	(+)	1	274	
Query_10077	(+)	1	138	
Query_10078	(+)	1	274	
Query_10079	(+)	1	137	
Query_10080	(+)	1	137	
Query_10081	(+)	1	136	
Query_10082	(+)	1	66	
Query_10083	(+)	1	274	
Query_10084	(+)	1	138	
Query_10085	(+)	1	137	
Query_10086	(+)	1	138	
Query_10087	(+)	1	137	
Query_10088	(+)	1	141	
Query_10089	(+)	1	274	
Query_10090	(+)	1	137	
Query_10091	(+)	1	138	
Query_10092	(+)	1	138	
Query_10093	(+)	1	273	
Query_10094	(+)	1	138	
Query_10095	(+)	1	138	
Query_10096	(+)	1	141	
Query_10097	(+)	1	137	
Query_10098	(+)	1	137	
Query_10099	(+)	1	138	
Query_10100	(+)	1	137	
Query_10101	(+)	1	140	
Query_10102	(+)	1	138	
Query_10103	(+)	1	138	
Query_10104	(+)	1	273	
Query_10105	(+)	1	279	
Query_10106	(+)	1	138	
Query_10107	(+)	1	139	
Query_10108	(+)	1	138	
Query_10109	(+)	1	274	
Query_10110	(+)	1	137	
Query_10111	(+)	1	137	
Query_10112	(+)	1	137	
Query_10113	(+)	1	138	
Query_10114	(+)	1	137	
Query_10115	(+)	1	78	
Query_10116	(+)	1	137	
Query_10117	(+)	1	138	
Query_10118	(+)	1	138	
Query_10119	(+)	1	138	
Query_10120	(+)	1	138	
Query_10121	(+)	1	138	
Query_10122	(+)	1	138	
Query_10123	(+)	1	138	
Query_10124	(+)	1	138	
Query_10125	(+)	1	137	
Query_10126	(+)	1	137	
Query_10127	(+)	1	274	
Query_10128	(+)	1	138	
Query_10129	(+)	1	138	
Query_10130	(+)	1	137	
Query_10131	(+)	1	278	
Query_10132	(+)	1	138	
Query_10133	(+)	1	146	
Query_10134	(+)	1	138	
Query_10135	(+)	1	138	
Query_10136	(+)	1	278	
Query_10137	(+)	1	137	
Query_10138	(+)	1	138	
Query_10139	(+)	1	274	
Query_10140	(+)	1	138	
Query_10141	(+)	1	138	
Query_10142	(+)	1	274	
Query_10143	(+)	1	138	
Query_10144	(+)	1	141	
Query_10145	(+)	1	138	
Query_10146	(+)	1	138	
Query_10147	(+)	1	138	
Query_10148	(+)	1	138	
Query_10149	(+)	1	138	
Query_10150	(+)	1	138	
Query_10151	(+)	1	138	
Query_10152	(+)	1	138	
Query_10153	(+)	1	138	
Query_10154	(+)	1	138	
Query_10155	(+)	1	138	
Query_10156	(+)	1	138	
Query_10157	(+)	1	138	
Query_10158	(+)	1	273	
Query_10159	(+)	1	138	
Query_10160	(+)	1	138	
Query_10161	(+)	1	138	
Query_10162	(+)	1	138	
Query_10163	(+)	1	274	
Query_10164	(+)	1	139	
Query_10165	(+)	1	274	
Query_10166	(+)	1	273	
Query_10167	(+)	1	138	
Query_10168	(+)	1	137	
Query_10169	(+)	1	137	
Query_10170	(+)	1	137	
Query_10171	(+)	1	137	
Query_10172	(+)	1	137	
Query_10173	(+)	1	137	
Query_10174	(+)	1	137	
Query_10175	(+)	1	137	
Query_10176	(+)	1	138	
Query_10177	(+)	1	137	
Query_10178	(+)	1	137	
Query_10179	(+)	1	137	
Query_10180	(+)	1	137	
Query_10181	(+)	1	137	
Query_10182	(+)	1	137	
Query_10183	(+)	1	137	
