# Supplementary material for: Insights into Functions of Universal Stress Proteins Encoded by Genomes of Gastric Cancer Pathogen Helicobacter pylori and Related Bacteria
Source: Pathogens. 2025 Mar 13;14(3):275. doi: 10.3390/pathogens14030275 (PMC11944479; doi:10.3390/pathogens14030275)
Supplement: Supplementary file 1 [file pathogens-14-00275-s001.zip › Supplementary-File-S7.pdf]

Universal Stress Protein Length	Gene Adjacency Transcription Direction Pattern among 1009 *Helicobacteraceae* Genes for Universal Stress Proteins<br>An example universal stress protein gene (in red) for each protein length and gene adjacency transcription pattern is shown.			
	**010**	**011**	**110**	**111**
137 aa	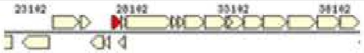<br>648251454 HPSAT_00150<br>*Helicobacter pylori* Sat464	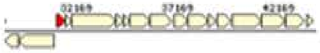<br>637017765 HP0031<br>*Helicobacter pylori* 26695	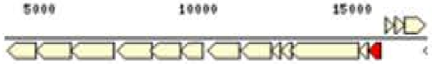<br>643093196 HP9810_897g16<br>*Helicobacter pylori* 98-10	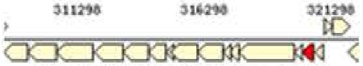<br>2535982025 HPCPY1313_1451<br>*Helicobacter pylori* CPY1313
138 aa	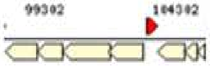<br>646265951 HWAG_01315<br>*Helicobacter winghamensis* ATCC BAA-430	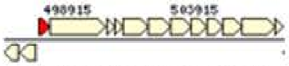<br>650934530 HBZC1_05260<br>*Helicobacter bizzozeronii* CIII-1	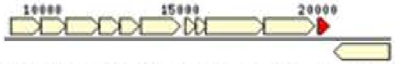<br>8032193790 Ga0347117_12_20668_21084<br>*Helicobacter pullorum* 35818_8	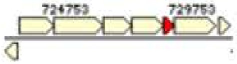<br>637431774 HH0744<br>*Helicobacter hepaticus* 3B1, ATCC_51449
139 aa				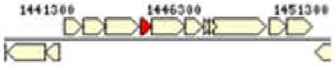<br>646683096 HMU13300<br>*Helicobacter mustelae* ATCC 43772
140 aa	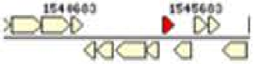<br>2702845445 Ga0112958_11781427<br>*Helicobacter saguini* MIT 97-6194	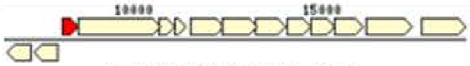<br>650063798 HSUHS1_0165<br>*Helicobacter suis* HS1		
141 aa		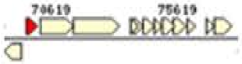<br>2563231197 HRAG_01292<br>*Helicobacter bilis* ATCC 43879	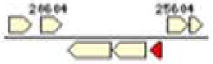<br>8032161046 Ga0347119_038_25390_25815<br>*Helicobacter trogontum* 50960_7	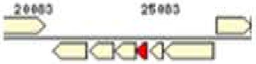<br>8032139321 Ga0347111_22_24869_25294<br>*Helicobacter muridarum* 216_8
146 aa				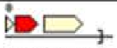<br>2996945265 Ga0337149_010_210_650<br>*Helicobacter didelphidarum* MIT 17-337
273 aa		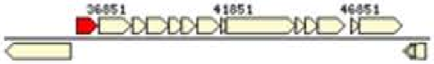<br>8056354404 Ga0616615_04_36439_37260<br>*Helicobacter turcicus* Faydin-H64	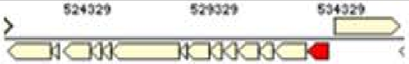<br>646265808 HWAG_01176<br>*Helicobacter winghamensis* ATCC BAA-430	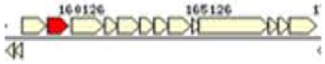<br>8032104691 Ga0337151_23_159714_160535<br>*Helicobacter ganmoni* MIT 99-5101
274 aa				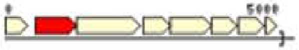<br>643921339_HcanM9_010100007916<br>*Helicobacter canadensis* MIT 98-5491, ATCC 700968
278 aa			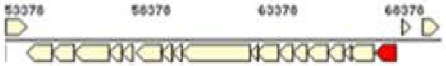<br>2989785770_Ga0339957_51_67958_68794<br>*Helicobacter burdigaliensis* CNRCH 2005/566H	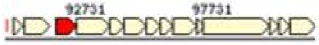<br>2996950993 Ga0336000_064_92311_93147<br>*Helicobacter valdiviensis* WBE14
279 aa				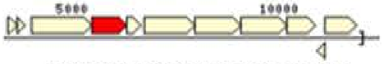<br>2789629299 Ga0302026_11666<br>*Helicobacteraceae* bacterium CG2_30_36_10
285 aa				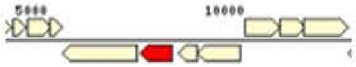<br>637455286 WS0007<br>*Wolinella succinogenes* DSM 1740
