# Supplementary material for: Insights into Functions of Universal Stress Proteins Encoded by Genomes of Gastric Cancer Pathogen Helicobacter pylori and Related Bacteria
Source: Pathogens. 2025 Mar 13;14(3):275. doi: 10.3390/pathogens14030275 (PMC11944479; doi:10.3390/pathogens14030275)
Supplement: Supplementary file 1 [file pathogens-14-00275-s001.zip › Supplementary-File-S9.pdf]

pathogens

MDPI

Article

Insights into Functions of Universal Stress Proteins Encoded by Genomes of Gastric Cancer Pathogen *Helicobacter pylori* and Related Bacteria

Authors: Raphael D. Isokpehi *, Shaneka S. Simmons, Angela U. Makolo, Antoinesha L. Hollman, Solayide A. Adesida, Olabisi O. Ojo * and Amos O. Abioye

* **Correspondence: raphael.isokpehi@outlook.com (R.D.I.); olabisi.ojo@asurams.edu (O.O.O.)**

Supplementary Tables and Figures

Table S1. *Helicobacter* genomes without species name encoding universal stress proteins grouped by anatomic niche and host organism.

Helicobacter Group by Anatomic Niche	*Helicobacter* Sub Group	Host Organism	*Helicobacter* Strain
Enterohepatic	EHS 1	Mouse	MIT 05-5294, MIT 11-5569
Enterohepatic	EHS 1	Pig	11-8110
Enterohepatic	EHS 3	Tortoise	11S02629-2, 13S00401-1
Enterohepatic	EHS 4	Mouse	MIT 14-3879
Enterohepatic	EHS 4	Iguana	16-1353
Enterohepatic	EHS 5	Mouse	MIT 03-1614, MIT 03-1616, 05-5293
Enterohepatic	EHS 6	Monkey	MIT 99-10781, MIT 00-7814
Enterohepatic	EHS 7	Monkey	MIT 01-3238
Enterohepatic	EHS 7	Human	CLO-3, HMC1
Enterohepatic	EHS 9	Lizard	11S02596-1, 11S03491-1, 12S02232-10, 12S02634-8, 13S00477-4, 13S00482-2
Enterohepatic		Human	106650
Enterohepatic		Opossum	15-1451
Enterohepatic		Human	TUL (“*Helicobacter caesarodunensis*” S15)
Gastric		Cat	NHP19-012
Gastric		Fox	L8

 https://doi.org/10.3390/xxxxx

Table S2. Lengths of universal stress proteins encoded by *Helicobacteraceae* genomes.

**Protein Length (aa)** [1]	***Helicobacteraceae* Genomes**
66, 68, 72, 78, 89, 99, 104, 111, 121, 126 and 136	14 *Helicobacter pylori* genomes.
88	*Helicobacter bilis* ATCC 51630
137	*H. acinonychis* (8 sequences), *H. cetorum* (3 sequences), and *H. pylori* (774 sequences)
138	180 non-*Helicobacter pylori Helicobacter* genomes and two *Wolinella succinogenes* genomes
139	Three strains of *H. mustelae* (ATCC 43772, NCTC 12031, and NCTC 12198) and *H. anatolicus* faydin-H8
140	Five strains of *H. sanguini* [MIT 97-6194, 15-1416 (F2), 16-048 (F4), 15-1458 (F3), and 97-6194-5 (F0)] and two strains of *H. suis* (HS1 and HS5)
141	22 genomes namely; *H. aurati* (2 strains), *H. bilis* (9 strains), *H. muridarum* (5 strains), *H. rappini* (1 strain), and *H. trogontum* (5 strains).
146	*H. didelphidarum* MIT 17-337
147	*H. pylori* GAM96Ai
273	*H. winghamensis* ATCC BAA-430, *H. rodentium* ATCC 700285, *Helicobacter* sp. MIT 05-5294, *Helicobacter* sp. MIT 11-5569, *H. ganmani* MIT 99-5101, and *H. turcicus* Faydin-H64
274	17 genomes namely, *H. apodemus* (2 strains), *H. canadensis* (2 strains, 3 genomes), *H. colisuis* (1 strain), *H. mesocricetorum* 87012_3, *H. pullorum* (9 strains), *Helicobacter* sp. 11-8110
278	*H. burdigaliensis* CNRCH 2005/566H and *H. valdiviensis* WBE14
279	The unclassified *Helicobacteraceae* strain CG2_30_36_10
285	Two strains of *Wolinella succinogenes* (DSM 1740 and NCTC11488).

[1] Protein lengths were collected from the Integrated Microbial Genomes and Microbiomes (IMG/M) system [1]. The 138 aa protein sequences were predicted from 35 *Helicobacter* species. In addition, 26 *Helicobacter* strains without species names were among the 138 aa sequences. The count of Helicobacter species associated with protein lengths 139 aa, 140 aa, and 141 aa were 2, 2, and 5, respectively (Table 3 and Figure S1). The genomes of *H. didelphidarum* MIT 17-337 and *H. pylori* GAM96Ai encoded USP sequences of lengths 146 aa and 147 aa respectively. The additional universal stress protein lengths and encoding *Helicobacter* species or strains are 273 aa (*H. ganmani, H. rodentium*, strain MIT 05-5294, strain MIT 11-5569, *H. turcicus* and *H. winghamensis*); 274 (*H. apodemus*, *H. candensis*, *H. colisuis*, *H. mesocricetorum*, *H. pullorum* and strain 11-8110); and 278 (*H. burdigaliensis* and *H. valdiviensis*). Only one USP sequence of 274 aa was encoded by the two strains of *H. apodemus* in the dataset. The two strains of *W. succinogenes* (DSM 1740 and NCTC11488) each has a second USP gene encoding a 285 aa protein sequence.

Table S3. Counts of *Helicobacteracae* universal stress protein sequence lengths according to gene transcription direction binary patterns.

	Gene Transcription Direction Binary Pattern [1]				
**Protein Length (aa)**	**010**	**011**	**110**	**111**	**Grand Total**
137	1	502	263	2	768
138	5	46	50	78	179
139				4	4
140	5	2			7
141		8	4	10	22
146				1	1
273		1	2	3	6
274				17	17
278			1	1	2
279				1	1
285				2	2
**Grand Total**	11	559	320	119	1009

[1] If the adjacent gene to the USP gene is in same gene transcription direction, “1” represents the gene transcription direction relative to the USP gene, otherwise “0”.

Genus_Species	Universal Stress Protein Length (aa)												
	137	138	139	140	141	146	147	273	274	278	279	285	Grand Total
Helicobacter acinonychis	8												8
Helicobacter ailurogastricus		6											6
Helicobacter anatolicus			1										1
Helicobacter anseris		1											1
Helicobacter apodemus									2				2
Helicobacter aurati					2								2
Helicobacter baculiformis		1											1
Helicobacter bilis					9								9
Helicobacter bizzozeronii		9											9
Helicobacter burdigaliensis		1								1			2
Helicobacter canadensis		3							3				6
Helicobacter canis		2											2
Helicobacter cetorum	3												3
Helicobacter cinaedi		37											37
Helicobacter colisuis		1							1				2
Helicobacter cynogastricus		1											1
Helicobacter didelphidarum						1							1
Helicobacter equorum		2											2
Helicobacter felis		22											22
Helicobacter fennelliae		6											6
Helicobacter ganmani		1						1					2
Helicobacter heilmannii		9											9
Helicobacter hepaticus		1											1
Helicobacter himalayensis		1											1
Helicobacter jaachi		1											1
Helicobacter japonicus		1											1
Helicobacter labetoulli		1											1
Helicobacter macacae		2											2
Helicobacter magdeburgensis		1											1
Helicobacter marmotae		2											2
Helicobacter mehlei		2											2
Helicobacter mesocricetorum		1							1				2
Helicobacter muridarum					5								5
Helicobacter mustelae			3										3
Helicobacter pullorum		9							9				18
Helicobacter pylori	774						1						775
Helicobacter rappini					1								1
Helicobacter rodentium		1						1					2
Helicobacter saguini				5									5
Helicobacter salomonis		6											6
Helicobacter sp.		26						2	1				29
Helicobacter suis		17		2									19
Helicobacter trogontum					5								5
Helicobacter turcicus		1						1					2
Helicobacter typhlonius		2											2
Helicobacter valdiviensis		1								1			2
Helicobacter vulpis		1											1
Helicobacter winghamensis		1						1					2
Helicobacteraceae bacterium											1		1
Wolinella succinogenes		2										2	4
Grand Total	785	182	4	7	22	1	1	6	17	2	1	2	1,030

Figure S1. Protein sequence lengths of 1030 universal stress proteins encoded in 1002 genomes from species of *Helicobacter* and *Wolinella* as well as an unclassified *Helicobacteraceae* bacterium. Among the 775 *H. pylori* genomes, universal stress protein encoded in the genome of *Helicobacter pylori* GAM96Ai has a unique length of 147 amino acids. The 138 aa sequences were predicted from two *Wolinella succinogenes* genomes and 180 non-*H. pylori Helicobacter* genomes (including enterohepatic and gastric helicobacters in Table 1 and Figure S1).

Cluster	Strain Group	Protein Length	Amino Acid Pattern	Genome Name	Gene ID USP
>Cluster 22	GAM	137	GISVIGSESHQ	Helicobacter pylori GAM42Ai	2537211452
				Helicobacter pylori GAM71Ai	2537213110
				Helicobacter pylori GAM80Ai	2537222220
				Helicobacter pylori GAM83Bi	2537223551
				Helicobacter pylori GAM83T	2537218845
				Helicobacter pylori GAM93Bi	2537219994
				Helicobacter pylori GAM100Ai	2536604317
				Helicobacter pylori GAM101Biv	2530669389
				Helicobacter pylori GAM103Bi	2530668125
				Helicobacter pylori GAM112Ai	2530664222
				Helicobacter pylori GAM114Ai	2530663657
				Helicobacter pylori GAM115Ai	2530661628
				Helicobacter pylori GAM117Ai	2542041744
				Helicobacter pylori GAM118Bi	2530659708
				Helicobacter pylori GAM119Bi	2530673040
				Helicobacter pylori GAM120Ai	2530671137
				Helicobacter pylori GAM121Aii	2536170229
				Helicobacter pylori GAM201Ai	2536172020
				Helicobacter pylori GAM210Bi	2536173965
				Helicobacter pylori GAM231Ai	2536175251
				Helicobacter pylori GAM239Bi	2536163724
				Helicobacter pylori GAM244Ai	2536164940
				Helicobacter pylori GAM245Ai	2536165951
				Helicobacter pylori GAM246Ai	2536168670
				Helicobacter pylori GAM249T	2536176389
				Helicobacter pylori GAM250AFi	2536178576
				Helicobacter pylori GAM250T	2531774057
				Helicobacter pylori GAM252Bi	2531772644
				Helicobacter pylori GAM252T	2531777037
				Helicobacter pylori GAM254Ai	2531775594
				Helicobacter pylori GAM260ASi	2531766658
				Helicobacter pylori GAM260Bi	2531765375
				Helicobacter pylori GAM263BFi	2531768757
				Helicobacter pylori GAM264Ai	2531780626
				Helicobacter pylori GAM265BSii	2531778864
				Helicobacter pylori GAM268Bii	2537214957
				Helicobacter pylori GAM270ASi	2537216365
				Helicobacter pylori GAMchJs106B	2537227231
				Helicobacter pylori GAMchJs114i	2531430853
				Helicobacter pylori GAMchJs124i	2531427439
				Helicobacter pylori GAMchJs136i	2531437831
		147	GISVIGSESHQ	Helicobacter pylori GAM96Ai	2537224544
>Cluster 118	GAM	137	GISVIGSESHQ	Helicobacter pylori GAM105Ai	2530666598
>Cluster 120	GAM	137	GISVIGSESHQ	Helicobacter pylori GAMchJs117Ai	2531429558
>Cluster 121	GAM	137	GISVIGSESHQ	Helicobacter pylori GAM260BSi	2531770876

Figure S2. Clusters, sequence lengths and amino acid pattern of functional sites for 45 universal stress proteins in four clusters of 45 strains of the *Helicobacter pylori* GAM strain group (Isolates from The Gambia, West Africa). All sequences share the same 11 functional sites (GISVIGSESHQ) for assessing ATP-binding. A noteworthy finding from the visualization is the common sequence length of 137 aa, except for strain GAM96Ai, which has 147 aa.

Pfam Domain	Protein Length										
	137	138	139	140	141	146	147	273	274	278	285
pfam00004===AAA		■		■							
pfam00106===adh_short					■						
pfam00733===Asn_synthase		■									
pfam00919===UPF0004					■						
pfam00994===MoCF_biosynth		■	■								
pfam01127===Sdh_cyt		■									
pfam01311===Bac_export_1		■									
pfam01936===NYN					■						
pfam02028===BCCT											■
pfam02617===ClpS	■						■				
pfam02646===RmuC			■								
pfam02861===Clp_N		■		■							
pfam03054===tRNA_Me_trans									■		
pfam03453===MoeA_N		■	■								
pfam03454===MoeA_C		■									
pfam03547===Mem_trans								■			
pfam03553===Na_H_antiporter					■						
pfam03772===Competence								■	■	■	
pfam04055===Radical_SAM					■						
pfam04341===DUF485		■									
pfam05437===AzlD		■									
pfam07724===AAA_2		■	■								
pfam07766===LETM1									■		
pfam07813===LTXXQ		■									
pfam08238===Sel1						■					
pfam10431===ClpB_D2-small		■		■							
pfam13181===TPR_8						■					
pfam13428===TPR_14		■									
pfam13481===AAA_25			■								
pfam13537===GATase_7		■									
pfam13541===ChlI			■								
pfam13726===Na_H_antiport_2					■						
pfam13729===TraF_2		■									
pfam15738===YafQ_toxin									■		
pfam17871===AAA_lid_9		■		■							
pfam18073===Rubredoxin_2			■								

Figure S3. Predicted protein domains encoded by genes adjacent and in same transcription direction to genes for universal stress proteins in *Helicobacter* and *Wolinella* genomes. Additional information on the pfam identifiers can be found at the InterPro website [2].

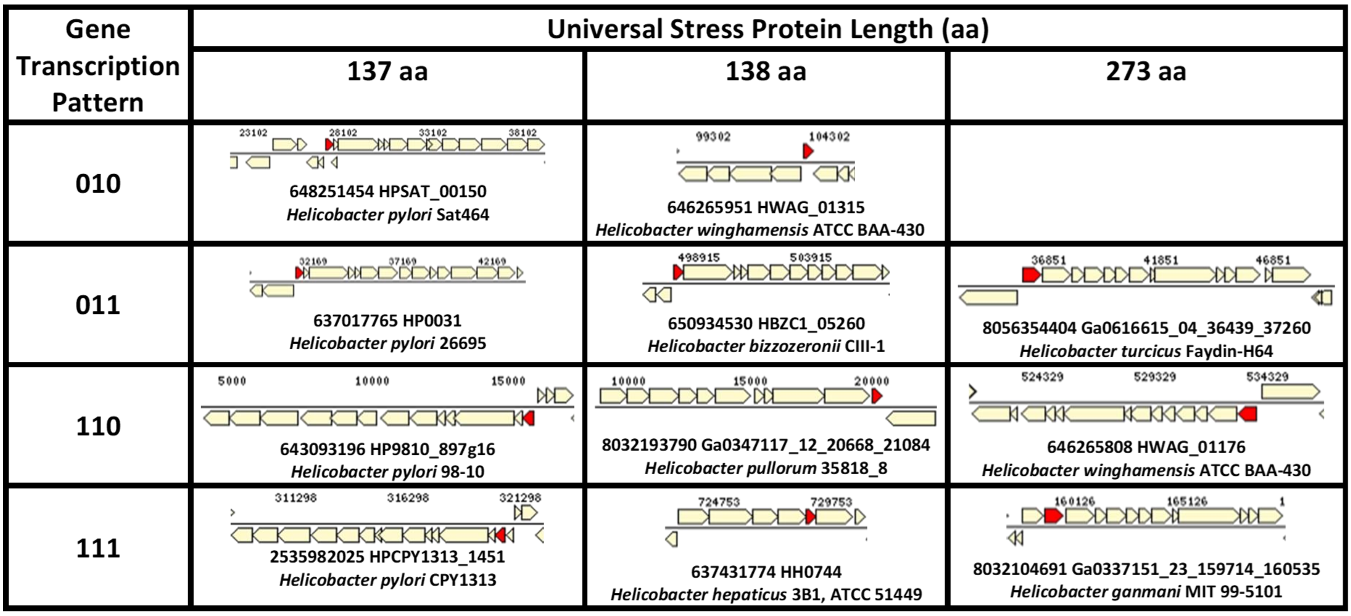

Figure S4. The gene transcription direction patterns (010, 011, 110 and 111) for three protein sequence lengths (137 aa, 138 aa and 273 aa) of selected *Helicobacter* universal stress proteins (USP). In the gene transcription pattern (three-digit binary number), the USP gene is assigned "1" and adjacent gene transcription direction is assigned "1" if same direction as USP gene, otherwise "0" is assigned. Each example of genomic context of USP gene is labelled with Gene ID, Locus Tag, and Genome Name obtained from the Integrated Microbial Genomes/Microbiomes (IMG/M). The patterns of gene transcription direction for strain Sat464 and strain CPY1313 are 010 and 111. These patterns are atypical compared with other *H. pylori* strains (110 or 011). In the case of Sat464, in the IMG/M genome annotation there is an adjacent upstream gene in opposite genome strand (IMG/M Gene ID: 648251455) to the USP Gene (IMG/M Gene ID: 648251454). The ClpS gene is on same strand as USP gene and has the IMG Gene ID of 648251456. For strain CPY1313, the upstream adjacent gene (IMG/M Gene ID: 2535982026) is in the same direction as the USP gene (IMG/M Gene ID: 2535982025). The IMG/M Gene ID for the ClpS gene is 2535982024.

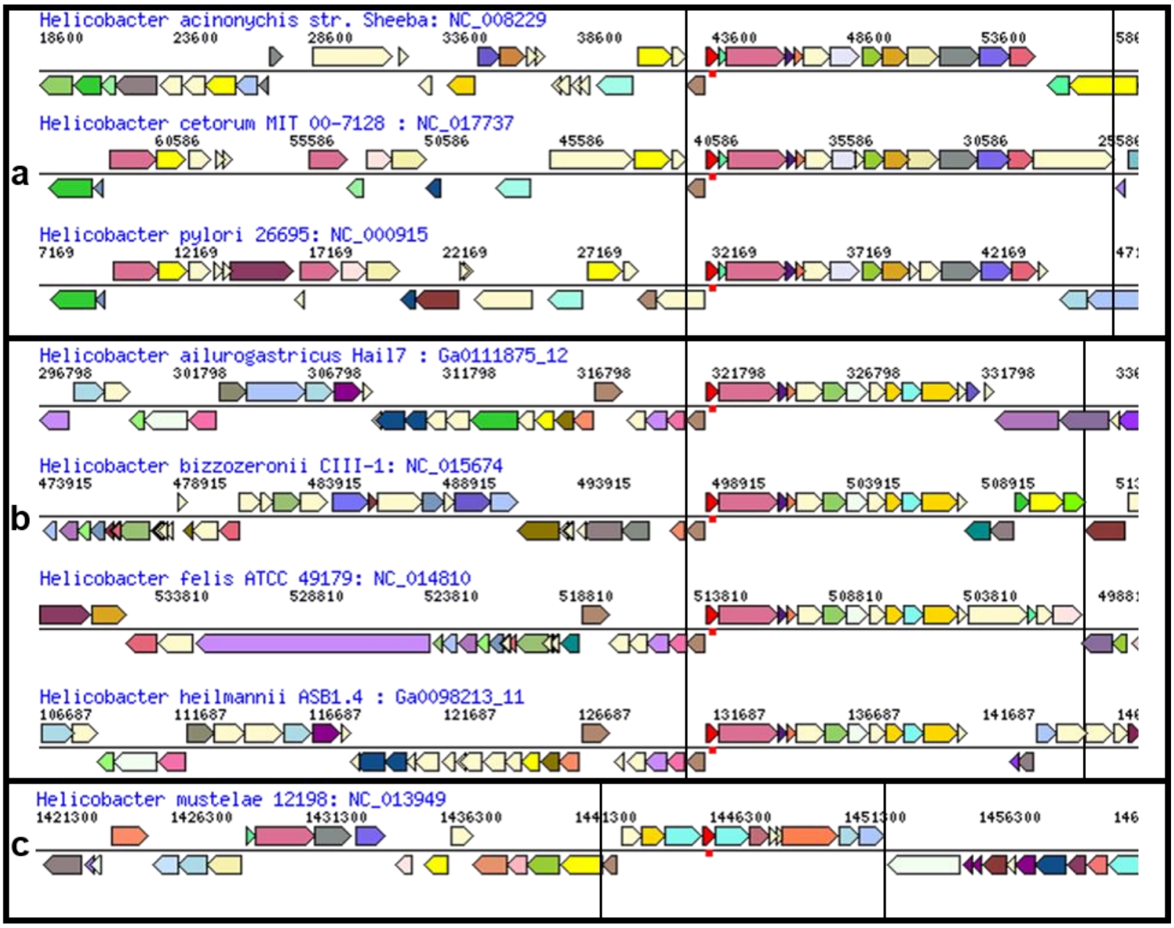

Figure S5. Integration of gene neighborhood diagrams of universal stress proteins genes (with red box) in genomes of selected helicobacters with an adjacent gene for ATP-dependent protease. Diagrams are from the Integrated Microbial Genomes/Microbiomes (IMG/M) website. Vertical lines within the genomic context provide boundaries that encompass the longest set of genes in the same direction as the USP gene for one of the genomes in the group. The figure provides a resource for comparative analysis of the gene neighborhoods of the selected USP genes. (a) The three gastric *Helicobacter* species (*acinonychis*, *cetorum* and *pylori*) have the gene for ATP-dependent Clp protease adapter protein ClpS. (b) The gene adjacent to the USP gene of three gastric helicobacters (*ailurogastricus*, *bizzozeronii*, *felis*, and *heilmannii*) does not encoded ClpS, but ATP-dependent Clp protease ATP-binding subunit ClpA. Other gastric helicobacters species with the same USP gene adjacency (not shown) are *baculiformis*, *cynogastricus*, *mehlei*, *salomonis*, *suis*, and *vulpis*. (c) The adjacent genes to the USP gene of *H. mustelae* are not predicted to encode ClpS or ClpA, but for an ATP-dependent serine protease and a DNA anti-recombination protein (rearrangement mutator).

References

1. Chen, I.-M.A.; Chu, K.; Palaniappan, K.; Ratner, A.; Huang, J.; Huntemann, M.; Hajek, P.; Ritter, S.J.; Webb, C.; Wu, D. The IMG/M data management and analysis system v. 7: content updates and new features. *Nucleic Acids Research* **2023**, *51*, D723-D732.
2. Paysan-Lafosse, T.; Blum, M.; Chuguransky, S.; Grego, T.; Pinto, B.L.; Salazar, G.A.; Bileschi, M.L.; Bork, P.; Bridge, A.; Colwell, L. InterPro in 2022. *Nucleic Acids Research* **2023**, *51*, D418-D427.

Disclaimer/Publisher's Note: The statements, opinions and data contained in all publications are solely those of the individual author(s) and contributor(s) and not of MDPI and/or the editor(s). MDPI and/or the editor(s) disclaim responsibility for any injury to people or property resulting from any ideas, methods, instructions or products referred to in the content.
