# Supplementary figures and images for: Insights into Functions of Universal Stress Proteins Encoded by Genomes of Gastric Cancer Pathogen Helicobacter pylori and Related Bacteria
Source: Pathogens. 2025 Mar 13;14(3):275. doi: 10.3390/pathogens14030275 (PMC11944479; doi:10.3390/pathogens14030275)

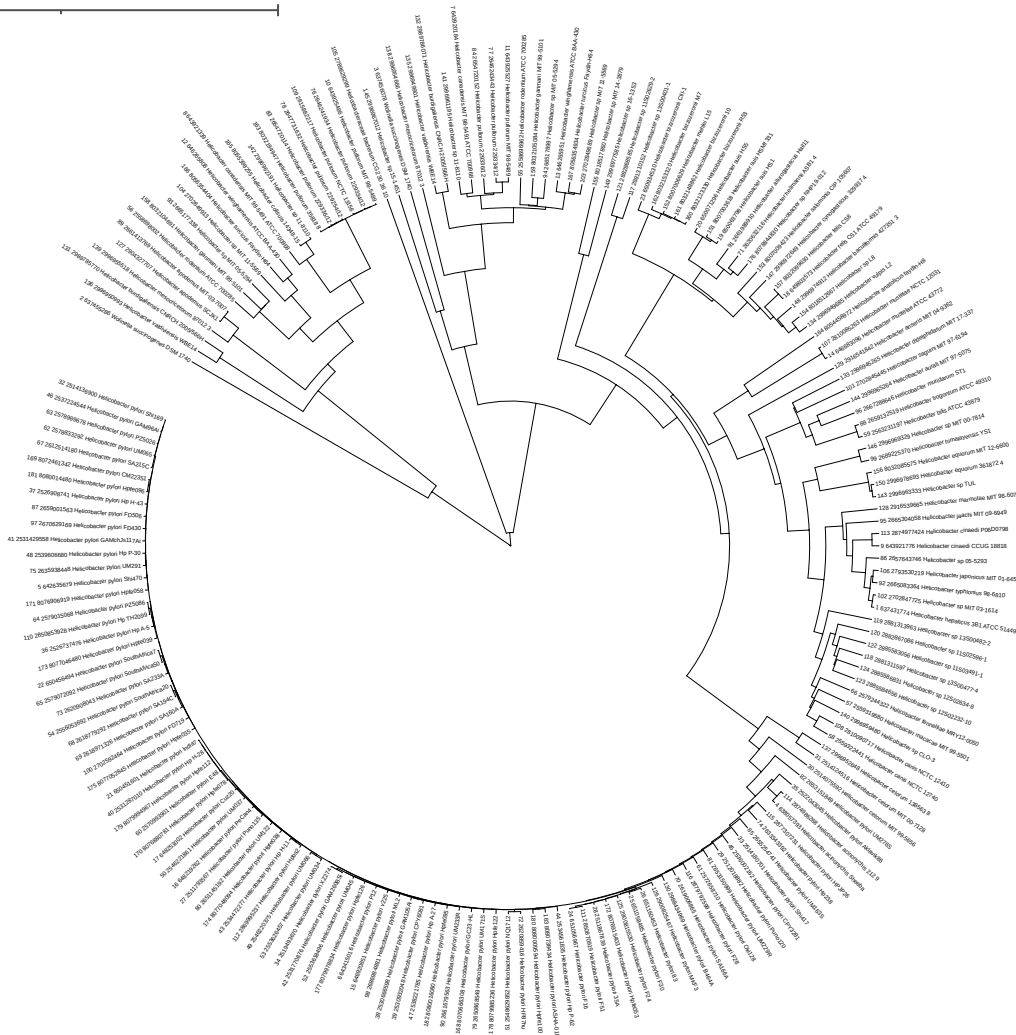

Supplement: Supplementary file 1 [file pathogens-14-00275-s001.zip › Supplementary-File-S6.pdf]
